# Impact of life history traits on gene flow: A multispecies systematic review across oceanographic barriers in the Mediterranean Sea

**DOI:** 10.1371/journal.pone.0176419

**Published:** 2017-05-10

**Authors:** Marta Pascual, Borja Rives, Celia Schunter, Enrique Macpherson

**Affiliations:** 1 Dept Genetics, Microbiology and Statistics/IrBio, Universitat de Barcelona, Diagonal 643, Barcelona, Spain; 2 KAUST Environmental Epigenetic Program (KEEP), Division of Biological and Environmental Sciences & Engineering and Division of Applied Mathematics and Computer Sciences, King Abdullah University of Science and Technology, Thuwal, Kingdom of Saudi Arabia; 3 Centre d’Estudis Avançats de Blanes (CEAB-CSIC), Car. Acc. Cala St. Francesc 14, Blanes, Girona, Spain; National Cheng Kung University, TAIWAN

## Abstract

**Background:**

Marine species can demonstrate strong genetic differentiation and population structure despite the hypothesis of open seas and high connectivity. Some suggested drivers causing the genetic breaks are oceanographic barriers and the species’ biology. We assessed the relevance of seven major oceanographic fronts on species connectivity while considering their dispersal capacity and life strategy.

**Methods:**

We systematically reviewed the scientific articles reporting population genetic differentiation along the Mediterranean Sea and across the Atlantic-Mediterranean transition. We retained those considering at least one sampling locality at each side of an oceanographic front, and at least two localities with no-front between them to correctly assess the effect of the front. To estimate the impact of life history characteristics affecting connectivity we considered the planktonic larval duration (PLD) and adult life strategy.

**Results:**

Oceanographic barriers in the Mediterranean Sea seem to reduce gene flow globally; however, this effect is not homogeneous considering the life history traits of the species. The effect of the oceanographic fronts reduces gene flow in highly mobile species with PLD larger than 2–4 weeks. Benthic sessile species and/or with short PLD (< 2 weeks) have more significant genetic breaks between localities than species with higher motility; however, genetic differentiation occurs independently of the presence of a front.

**Conclusion:**

Genetic connectivity is important for populations to recover from anthropogenic or natural impacts. We show that species with low mobility, mostly habitat-formers, have high genetic differentiation but low gene flow reduction mediated by the front, therefore, considering the importance of these species, we emphasize the vulnerability of the Mediterranean ecosystems and the necessity of protection strategies based on the whole ecosystem.

## Introduction

The Mediterranean Sea displays one of the world’s richest diversity [1]. This basin contains more than 20,000 species of fish, cetaceans, invertebrates, sea turtles, algae and seaweeds, with a large proportion of endemism (ca. 20%). For this reason the Mediterranean Sea has been defined as a biodiversity hotspot for conservation priorities. As a consequence, there is an increasing interest to protect this unique natural heritage, and several Congress and World Summits have called on countries to establish a system of networks of marine protected areas (MPAs) with the aim of covering 20 to 30% of the total area [2]. Furthermore, an emphasis is laid on commercial species, where the management structures have not been sufficiently enforced [3].

Modelling studies have indicated the great importance of spatial configurations of MPAs and stock identification units to promote population and ecosystem persistence [4,5]. Although these configurations are clear in modelling results, efforts to assess and design MPAs and stock identifications are hindered by the lack of knowledge of several important factors. One of the major issues is the uncertainty about propagule dispersal which is one of the essential processes connecting areas and populations [6–8]. Currents and other oceanographic processes, which are often not being considered in management policies, potentially influence this dispersal capability and connectivity [9,10].

The majority of marine animals show life histories characterized by a long planktonic larval phase potentially allowing long-distance dispersal by marine currents. Therefore, we would expect to see low genetic structuring between localities of many marine species [11,12]. This could be particularly true in species with high fecundity or very large population sizes that potentially can have long-distance dispersal of eggs, larvae or adults [13]. However, a large number of studies in the last decade have disproven the concept that the seas are ‘open’ and well–connected and it has been shown that a number of species present a spatial genetic differentiation which is higher than expected if we only consider their dispersive abilities [14–16]. Several mechanisms may cause genetic differentiation between populations such as vicariance processes, caused by historical barriers, oceanographic currents, habitat discontinuities, local adaptation, larval behaviour, isolation by distance and limited dispersal capabilities [17]. Therefore, the global level of genetic differentiation within species results from a complex equilibrium between structuring factors (e.g. oceanographic fronts, isolation by distance) and homogenising factors (e.g. long larval pelagic phase, migratory behaviour of adults) [11].

The length of the pelagic phase or planktonic larval duration (PLD) could be considered as proxy of the species’ dispersal potential, being one of the most important homogenising factors in the population structure [18]. In the marine environment, dispersal can be validated using genetic markers as a measure of connectivity among localities [19]. Some studies have demonstrated that PLD and genetic metrics typically reflect scales of dispersal [20] but see the review of Selkoe et al.[9], whereas others have shown no clear patterns between genetic connectivity and dispersal capabilities [21].

**Fig 1:**
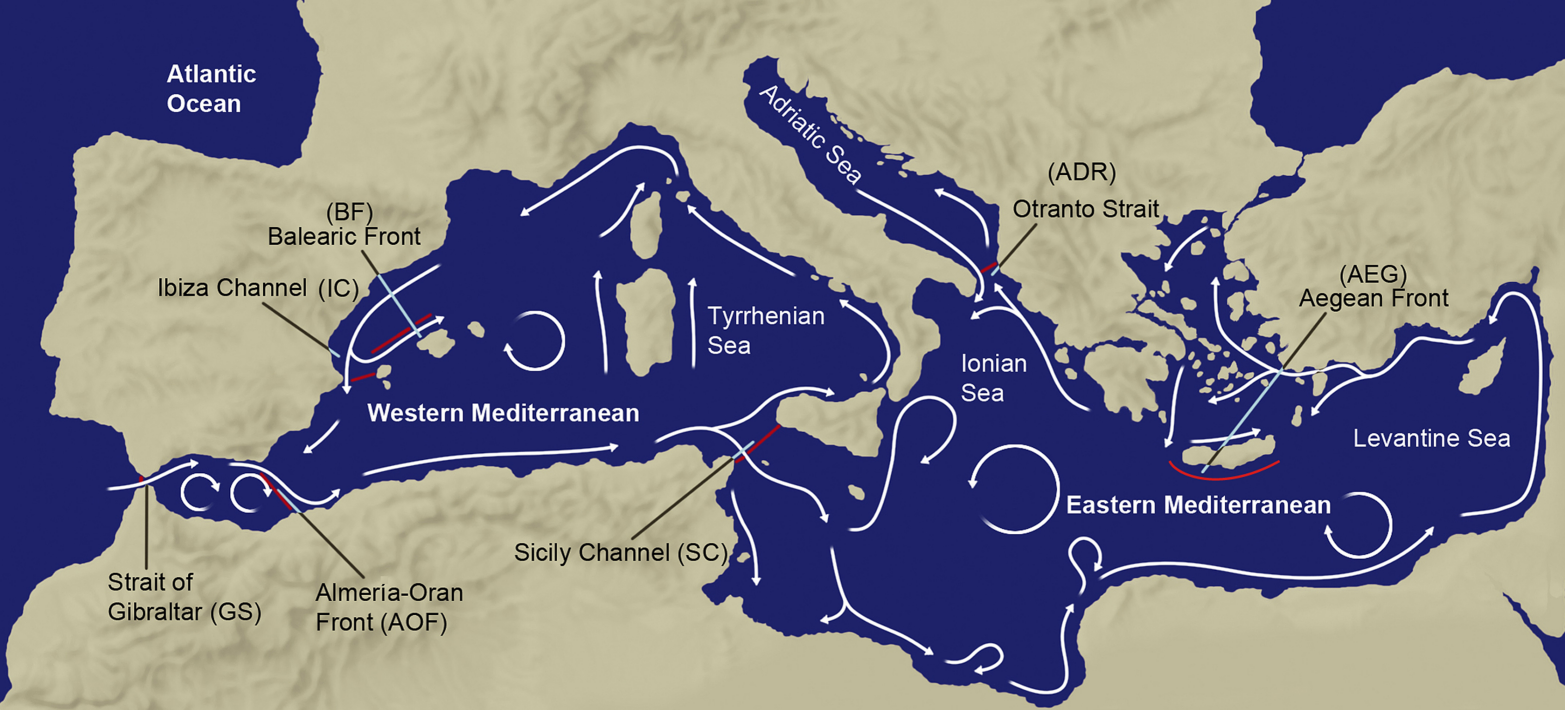
Map of the Mediterranean Sea with the name of the sub-basins, main currents (white lines) and oceanographic fronts analysed (red lines). The name of the fronts and the acronym used (in black) is as follows: GS (Gibraltar Strait), AOF (Almeria-Oran Front), IC (Ibiza Channel), BF (Balearic Front), SC (Sicily Channel), ADR (Otranto Channel), AEG (southern margin of the Aegean Sea).

In the Mediterranean Sea, oceanographic processes, such as current patterns and oceanographic discontinuities are crucial factors influencing population genetic connectivity [17,21,22]. Hence, to understand connectivity patterns on a large-scale it is important to consider the physical processes possibly influencing gene flow between localities. The Mediterranean Sea is an ideal study area for a survey incorporating oceanographic features and gene flow. The circulation patterns within the Mediterranean Sea is well described [23,24]. Moreover, several oceanographic discontinuities (Fig 1), mostly on the Spanish coast, originated by the entry of less saline Atlantic waters throughout the Gibraltar Strait have been reported to act as barriers to gene flow for numerous species [21,25,26]. The best-studied discontinuity is the Almeria-Oran Front (AOF), which has been proposed to be the main point of genetic break between the Atlantic Ocean and the Mediterranean Sea [27]. Other fronts have also shown to be strong barriers for genetic exchange in some species, such as the Balearic Front (BF) [21] or the Ibiza channel (IC) [28]. Other oceanographic processes occurring within the Mediterranean Sea, e.g. along the Sicily Channel, the Otranto Channel or the southern margin of the Aegean Sea, can also act as barriers to gene flow, however, their importance is not well studied [29–31].

Recently, some studies have provided interesting models defining hydrodynamic provinces, by coupling Lagrangian simulations of particles with oceanographic currents to modelate the transport of larvae in order to characterize marine connectivity [32,33]. The main aim of these models was to identify natural conservation units to be used in the establishment of an MPA network. These units would be defined as the Cells of Ecosystem Functioning (CEF), based on both oceanographic and ecological processes, arranged in space so as to account for both patterns (biodiversity distribution) and processes (ecosystem functioning) [34]. However, these CEFs do not include data on gene flow among localities hence lacking an important factor in connectivity processes. A review by Patarnello et al. [27] was the first attempt to summarize the genetic data regarding the biogeographical separation between the Mediterranean and Atlantic biota. This review (including 20 studies) showed steep changes of allele frequencies associated with the Almeria-Oran Front (AOF) but failed to relate biological traits with genetic differentiation. After this review numerous genetic studies have been carried out in different areas of the Mediterranean, most of them analysing one or more oceanographic fronts. Some of the studies, however, do not provide data comparing localities separated by fronts and control sites to correctly evaluate the influence of the front. Other studies contain genetic differences between sampling localities that include more than one front between them making it difficult to draw adequate conclusions on the influence of the oceanographic fronts on gene flow. Hence, there is a lack of a comprehensive analysis on the effect of oceanographic fronts on gene flow in the Mediterranean Sea, which can seriously compromise our knowledge on the connectivity patterns among localities and in the establishment of MPA networks [34].

In the present review we aim to assess the effect of life history characteristics on population differentiation considering the impact of oceanographic discontinuities. In order to correctly evaluate whether a front is the cause for genetic differences between localities we screened all population genetic studies based in the Mediterranean Sea considering at least one sampling locality at each side of the oceanographic front, and at least two localities with no-front between them. We focus our review on the seven most important Mediterranean oceanographic discontinuities: Gibraltar Strait (GS), Almeria-Oran Front (AOF), Ibiza Channel (IC), Balearic Front (BF), Sicily Channel (SC), Otranto Channel (ADR) and the southern margin of the Aegean Sea (AEG) [23,35,36]. Our main goal is to evaluate the impact of life history characteristics on connectivity reduction, in both the absence and presence of fronts, so we categorized all species based on their larval dispersal capabilities and their adult behaviour. Finally, we discuss the potential implications of life history traits and oceanographic discontinuities in the establishment of management units and networks of MPAs.

## Materials and methods

**Fig 2:**
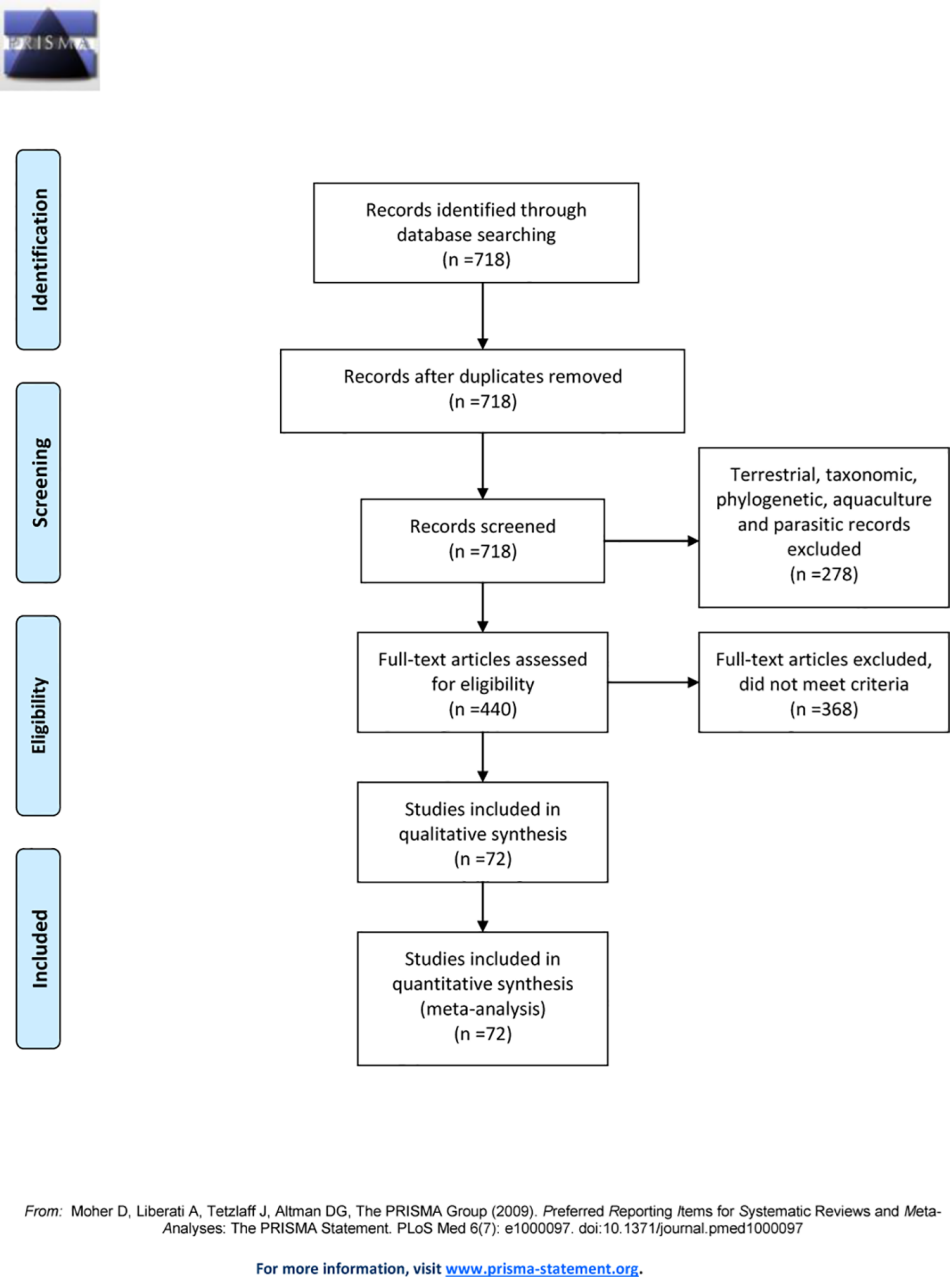
PRISMA flow diagram for literature search.

We searched the ISI Web of Knowledge database for scientific articles dated until 2016 which evaluated population genetic differentiation along the Mediterranean Sea and across the Atlantic-Mediterranean transition. Keywords were selected to identify these studies: “Mediterranean Sea”, “gene flow”, “genetic/population structure”, “genetic differentiation”, “connectivity”, which resulted in a total of 718 studies (Fig 2). We assessed 440 papers but retained only 72 that could be used to correctly evaluate the impact of oceanographic fronts (Fig 2 and S1 Checklist). We retained only those studies meeting the following criteria: reporting the analysis of genetic differentiation between localities situated at both sides of one front and including genetic data from localities not separated by a front that could be used as control, and also based on molecular marker selection see below. A comparison was also excluded whenever more than one front was present between two localities. Control localities are necessary to correctly evaluate the effect of a front as they allow discriminating between reduced connectivity due to the species limited dispersal capabilities or to the effect of the oceanographic front [21]. Invasive species were not included in the analyses to avoid confounding effects due to genetic differentiation between localities driven by passive colonization, sometimes from different native areas and highly influenced by genetic drift during arrival of colonisers [37–39].

### Molecular markers selection

Different molecular markers have been suggested to be more adequate at identifying historical (e.g. mtDNA) or contemporary (e.g. microsatellites) processes [40]. We therefore classified marker types into two groups: Mitochondrial genes, including COI, 16S, cyt b were categorized as “MT”, and nuclear polymorphic genes including allozymes, microsatellites and SNPs under the category “NUC”. For some species more than one study or analysis with several different molecular markers and genetic distance measurements were available. To avoid biases due to overrepresentation of the same species in each front, only one analysis per species per front was considered. The following criteria were applied: (1) for studies applying nuclear and mitochondrial markers the former were preferentially chosen; (2) when several indices were reported we preferentially retained the data for F_ST_ values between localities against other less frequently used indices.

### Oceanographic discontinuities

Between the Atlantic Ocean and Mediterranean Sea there are 7 major fronts frequently analysed in population genetic and biogeographic studies (see Fig 1): Gibraltar Strait (GS), Almeria-Oran (AOF), Ibiza Channel (IC), Balearic Front (BF), Sicily Channel (SC), Otranto Channel (ADR), and the southern margin of the Aegean Sea (AEG). The inflow of the North Atlantic Central Water (NACW) throughout the Gibraltar Strait (GS) is the most important oceanographic process between the Atlantic Ocean and Mediterranean Sea [36,41]. The GS discontinuity is located just before the entry of the Atlantic waters throughout the Gibraltar Strait, when the NACW recirculates near the Strait, in front of Cape Trafalgar towards the northwest along the coast of Cadiz [42]. Once in the Mediterranean Sea the NACW encounters the higher density Mediterranean water generating the Almeria-Oran front (AOF), a quasi-continuous front where its northern end detaches from the Spanish coast between Almeria and Cartagena and its southern end terminates around Oran on the North African coast [43]. The NACW is modified, increasing its salinity, into a mass usually called Modified Atlantic Water (MAW) that reaches the north-western Mediterranean basin and is deflected eastward by the cyclonic circulation around the Balearic Islands forming a well-defined second density front, the Balearic Front (BF). Just south of this front is the Ibiza Channel (IC), 80 Km width and 800 m depth, corresponding to the passage intersecting the Balearic topographic ridge between Ibiza and the Iberian Peninsula at Cape La Nao (Fig 1). The MAW also flows along the Algerian coast and crosses the Sicily Channel (SC), which divides the Western and Eastern basins of the Mediterranean Sea [35,36]. The next front considered going eastwards is the Otranto Channel (ADR) located at the entrance of the Adriatic Sea whose sill is 800 m deep [44]. Finally, around the southern margin of the Aegean Sea (AEG) another oceanographic discontinuity is observed formed by several cyclonic, anticyclonic gyres and eddies interconnected by currents and jets flowing at speeds of 20–30 cm s^-1^ [45].

### Life history data

To evaluate the impact of life history characteristics affecting connectivity we considered the planktonic larval duration (PLD), as a proxy of larval dispersal, and adult life behaviour. Each species was assigned to one of the following three groups according to their PLD: 1–15 days (S), 16–30 days (M), ≥31 days (L). Another categorical variable was constructed according to adult life strategy (LIFE): species were considered to be benthic sessile or with limited motility (BS), benthic vagile (BM), or pelagic (PEL). Finally, we also categorized the species according to major taxonomic groups: Angiosperm, Porifera, Cnidaria, Echinodermata, Mollusca, Crustacea, Tunicata and Pisces and grouped into a phylogenetic tree [46]. Biological information was obtained from the literature for angiosperm [47], porifera [48], cnidaria [49], echinodermata [50], mollusca [51–53], crustacea [54], tunicata [55] and pisces [56,57] (see also references cited in these articles).

### Statistical analyses

Pairwise genetic population distances (preferentially F_ST_), comparing localities separated by fronts and one control pairwise comparison representative of no-fronts, were extracted from the selected scientific articles. Geographic distances between locations were approximated following the coastline with Google Earth.

For different life history categories (PLD and LIFE strategy) we tested differences in frequency of significant genetic distances between localities, separated by fronts or control sites, by chi-square tests [58].

In order to avoid differences in significance due to isolation by distance, for each selected article all pairwise genetic distances (F_ST_) were also standardized by geographic distance in Km as in Galarza et al.[21]. We considered the existence of connectivity reduction to be mediated by the front when the standardized F_ST_ between localities separated by front was larger than between control sites. We compared the two standardized pairwise F_ST_ (with front and no-front) with a Wilcoxon matched pairs test for each variable (FRONT, PLD, LIFE strategy and MARKER) using the programme STATISTICA V 8 [59].

To analyse the interaction between the front effect and the other variables, we created a continuous variable ranging between 0 and 1 called NDIF. This variable was calculated as (x-xmin) / (xmax-xmin) were x represents the difference between front and no-front standardized F_ST_ values, xmax is the highest difference value and xmin the smallest one. We performed permutational multivariate analysis of variance (PERMANOVA) using the statistical package PRIMER-E v6 [60]. We used NDIF as dependent variable and PLD (S, M, L), LIFE strategy (BS, BM, PEL), MARKER (MT, NUC) and FRONT (Gibraltar Strait (GS), Almeria-Oran (AOF), Ibiza Channel (IC), Balearic Front (BF), Sicily Channel (SC), Otranto Channel (ADR), and the southern margin of the Aegean Sea (AEG)) as fix factors. PLD and LIFE strategy were not analysed together because not all levels were represented in both predictive variables. The assayed interactions included FRONT×PLD, FRONT×LIFE, and FRONT×MARKER. For each analysis 999 permutations were carried out.

## Results

In total 72 scientific papers passed our requirements to assess the effect of the fronts along the Mediterranean Sea and across the Atlantic-Mediterranean transition. These papers measured genetic differentiation between localities influenced by a front as well as a control comparison between localities without no-front (S1 Table). The mean geographic distance (±SE) between the selected control locations was 305 Km (± 13.3 Km). Fronts were evaluated selecting two localities on each side of the front with a mean distance of 492 Km ± 25.2 Km. Overall, we had information of 176 datasets of 70 species analysing different fronts (S2 Table). Fishes were the most represented taxonomic group (41.1%), followed by crustaceans (21.0%) and molluscs (13.6%). The mean (±SE) number of fronts correctly analysed per species in each reference was 2.12 ± 0.13.

### Genetic data and life history traits

**Fig 3:**
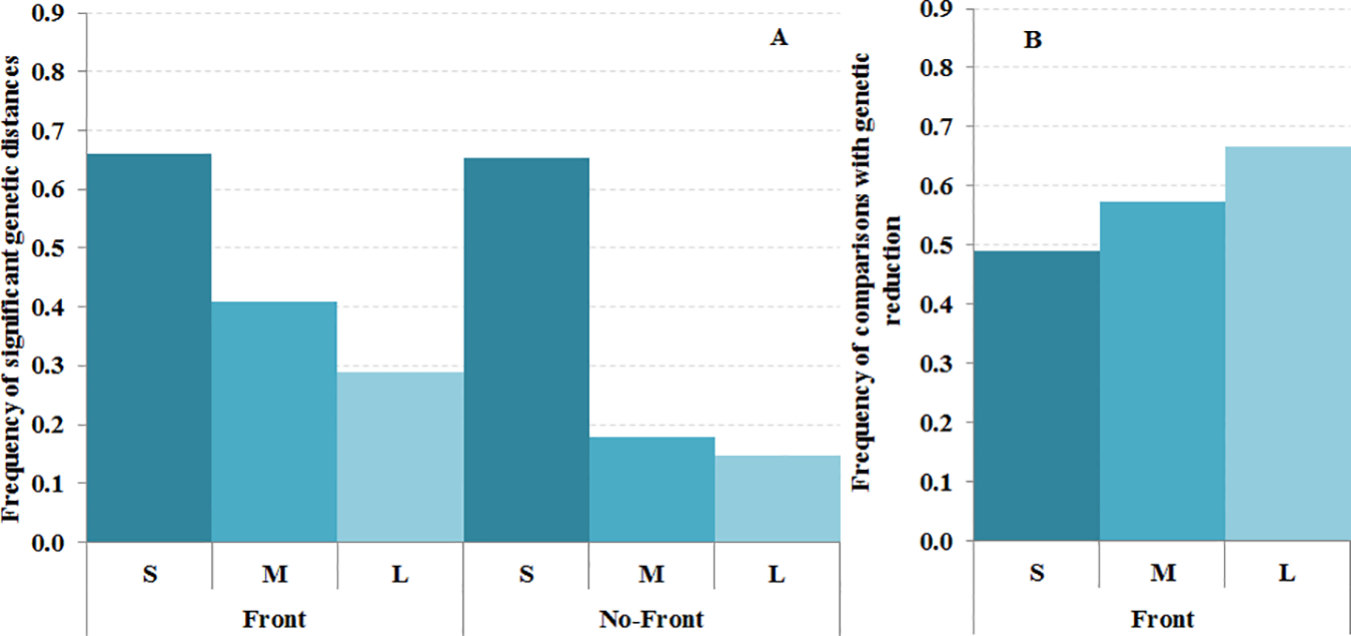
Influence of Planktonic larval duration (PLD) categories on genetic connectivity. (A) Frequency of significant genetic differences (P<0.05) between localities separated by a front and without a front. (B) Frequency of comparisons showing genetic reduction mediated by the front. PLD categories are identified as S = 1–15 days, M = 16–30 days and L ≥ 31 days.

**Fig 4:**
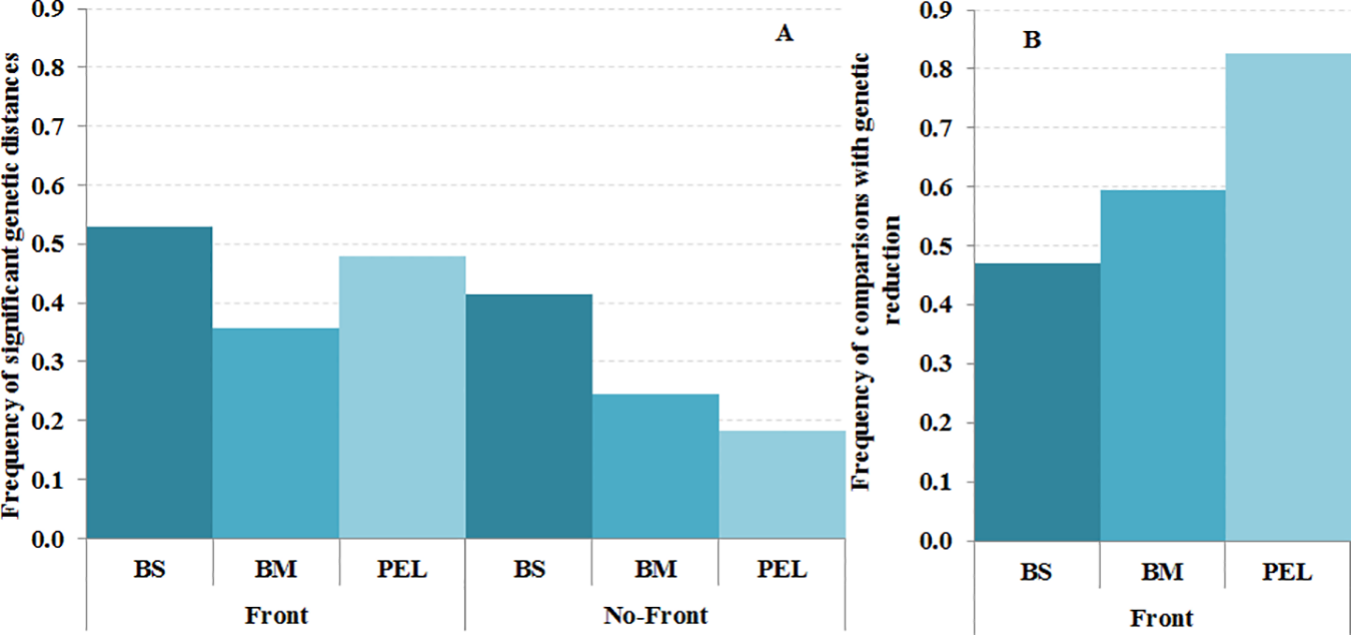
Influence of adult LIFE strategy categories on genetic connectivity. (A) Frequency of significant genetic differences (P<0.05) between localities separated by a front and without a front. (B) Frequency of comparisons showing genetic reduction mediated by the front. LIFE strategy categories are identified as BS = Benthic sessile or limited motility, BM = benthic vagile and PEL = pelagic.

We observed that 66% of comparisons involving species with short PLD (S) had significant differences between localities, while only 41% and 28% were significant for species with medium (M) and long (L) PLD, respectively (Fig 3A). This difference was observed in sites separated by a front (Chi: 16.53, p = 0.0003) and also in control sites not separated by any known front (Chi: 18.66, p = 0.0001). A similar trend was observed considering the LIFE strategy variable, where the percentage of significant comparisons reduced with increased mobility (Fig 4A). The percentage of benthic sessile (BS) species showing significant genetic differences was always higher than in species with higher motility capacity (BM and PEL). However, no significant differences between categories were observed for locations separated by a front (Chi: 4.33, p = 0.1146) or without front (Chi: 2.78, p = 0.2490).

### Effects of oceanographic fronts

We compared the standardized F_ST_ distances between localities separated by the front (F) and those without front (NF) and considered gene flow reduction when the former distance was larger. The frequency of comparisons with reduction increased with PLD (Fig 3B), being 49% for species with small PLD and 66% for species with large PLD. The same tendency was observed for adult LIFE strategy (Fig 4B), with benthic sessile or limited motility species showing the lowest number of comparisons with front reduction (47%) and pelagic species the largest (82%).

**Fig 5:**
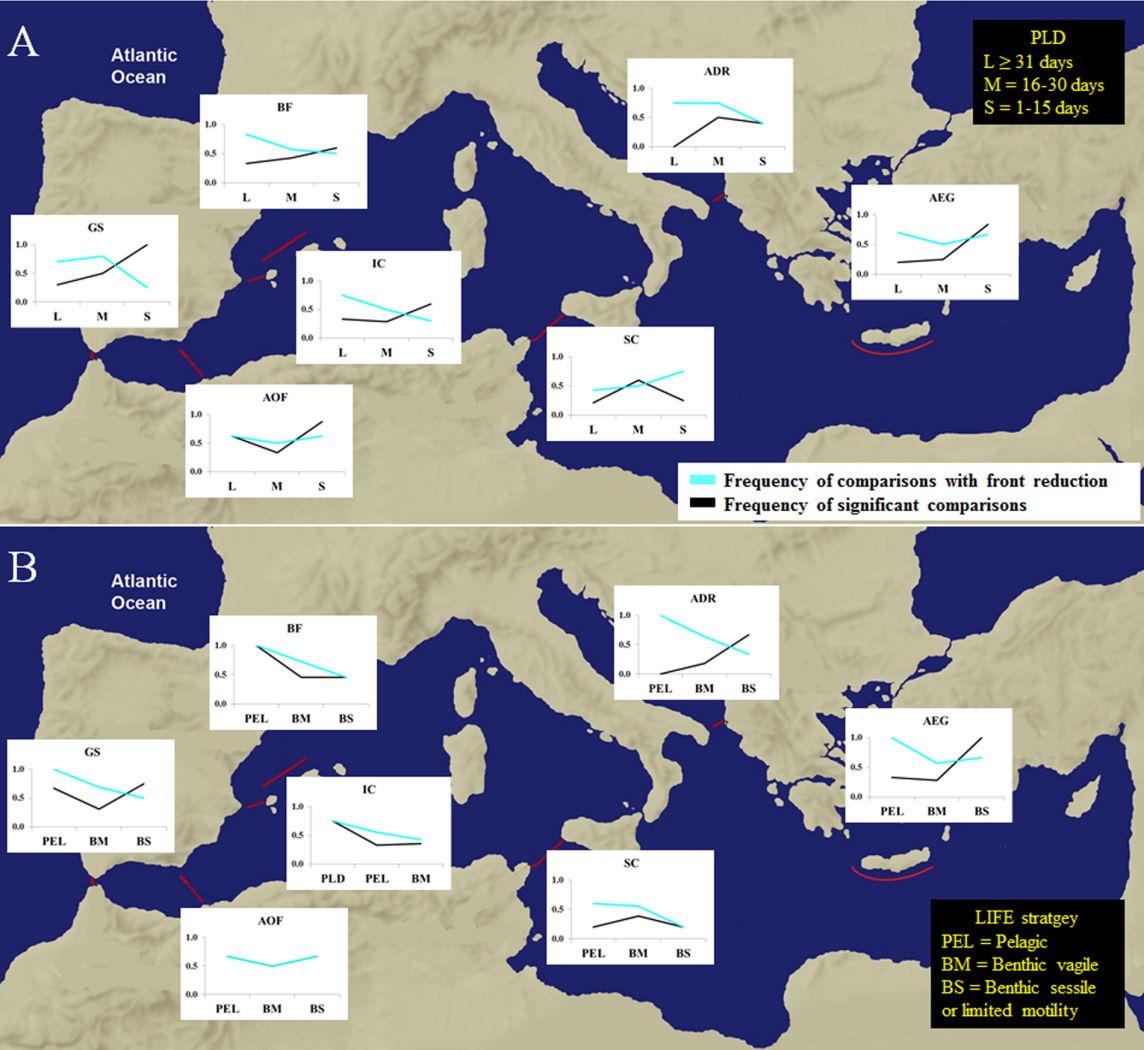
Genetic differentiation patterns between localities separated by oceanographic fronts. Frequency of species, for each PLD category (A) and LIFE strategy (B), showing significant genetic distances (black) and gene flow reduction (blue) between localities separated by oceanographic fronts. Each oceanographic front (red lines) is analysed separately: Gibraltar Strait (GS), Almeria-Oran Front (AOF), Ibiza Channel (IC), Balearic Front (BF), Sicily Channel (SC), Otranto Channel (ADR), and the southern margin of the Aegean Sea (AEG).

We also evaluated the impact of life history traits (PLD, LIFE strategy and the two of them combined to integrate the whole life-history) on the connectivity reduction mediated by fronts using Wilcoxon matched pairs tests (Table 1). Interestingly, oceanographic discontinuities significantly reduced gene flow in species with L PLD, with PEL LIFE strategy, as well as in species with BMM and PELL categories combining both adult and larval mobility capacities (Table 1). No significant reduction mediated by the front was observed with Wilcoxon matched pairs test for different types of markers (Table 1). Thus, although presenting higher genetic differentiation between localities (Figs 3A and 4A), species with low dispersal capabilities seemed less affected by the fronts (Figs 3B and 4B). On the contrary, the connectivity of species with larger motility capacity would be more affected by oceanographic discontinuities. We detected gene flow reduction for species with long PLDs (more than two weeks) in most Mediterranean fronts (Fig 5 and S3 Table). Furthermore, higher frequency of significant genetic differentiation in short PLD species and reduced adult mobility across discontinuities was observed across most Mediterranean oceanographic fronts (Fig 5 and S3 Table).

**Table 1:** Wilcoxon matched pairs test comparing the standardized F_ST_ distances between localities separated by fronts (F) and those without front (NF) for different larval and adult stage categories, and type of molecular marker.

Variable	Categories	n	NF>F	NF<F	Z	p
	S	47	24	23	1.25	0.212
PLD	M	61	26	35	1.46	0.144
	L	68	23	45	2.54	**0.011**
	BS	53	28	25	1.00	0.315
LIFE strategy	BM	101	40	60	1.89	0.058
	PEL	22	4	18	2.52	**0.012**
LIFE-PLD COMBINED	BSS	29	17	12	1.72	0.086
	BSM	17	9	8	0.02	0.981
	BSL	7	2	5	1.35	0.176
	BMS	18	7	11	0.11	0.913
	BMM	41	15	26	2.22	**0.026**
	BML	42	19	23	0.64	0.521
	PELM	3	2	1	0.53	0.593
	PELL	19	2	17	3.54	**0.000**
MARKER	MT	75	30	45	1.09	0.275
	NUC	101	43	58	0.73	0.465

PLD (1–15 days (S), 16–30 days (M), ≥31 days (L)), LIFE strategy (Benthic sessile or limited motility (BS), benthic vagile (BM), pelagic (PEL)), LIFE-PLD COMBINED (integrating adult strategy, LIFE, and larval mobility, PLD, in one variable), and MARKER (nuclear (NUC) and mitochondrial, (MT) DNA). NF>F indicates that the gene flow is larger between localities not separated by a front and NF<F indicates the contrary. n = number of comparisons. Significant p values in bold.

**Fig 6:**
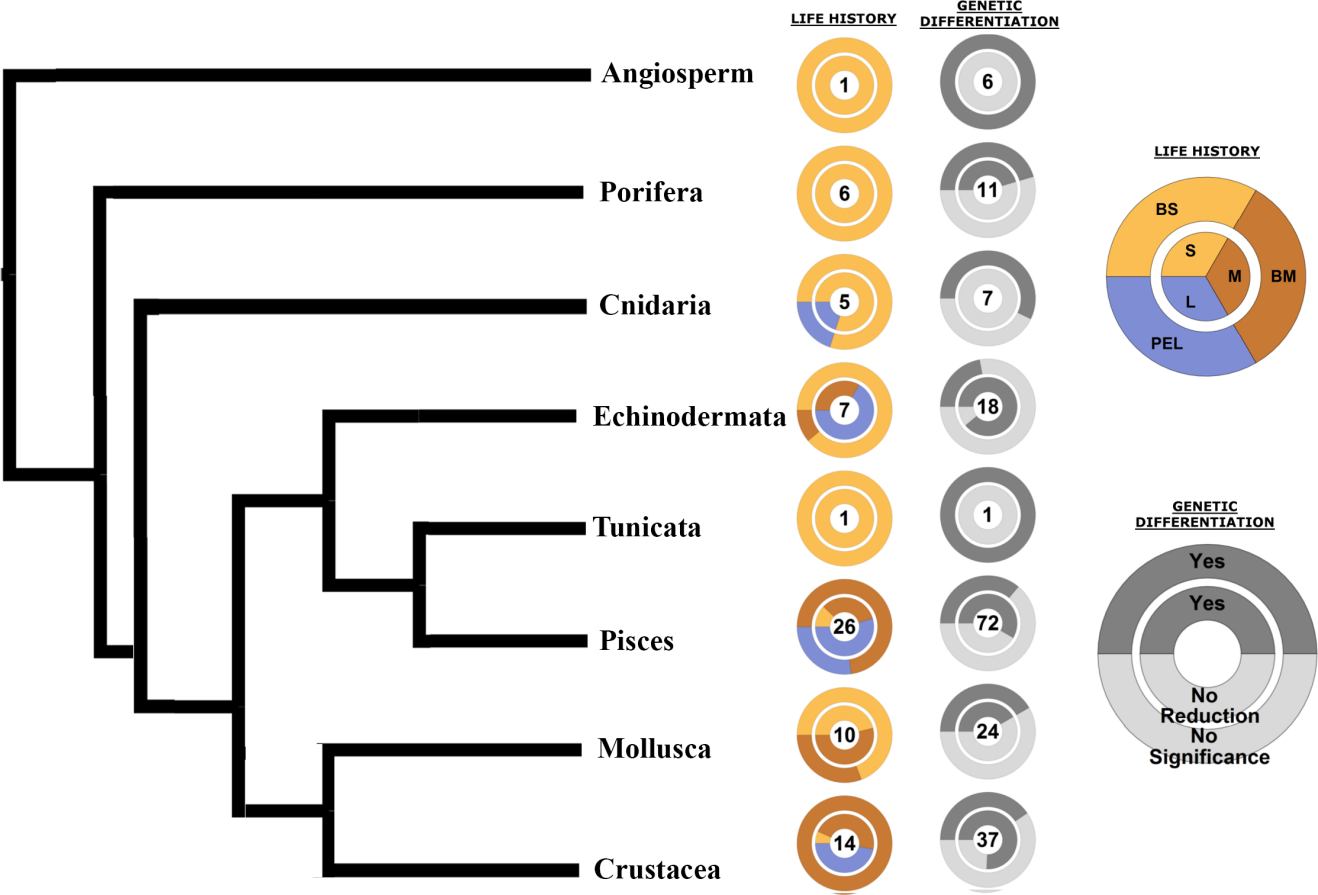
Phylogenetic tree of analysed taxa representing life history traits and genetic differentiation patterns. In the Life History column the number of species is given for each taxon, with the inner circle representing PLD categories (S = Orange, M = Brown, L = Blue) and the outer circle representing LIFE strategy categories (BS = Orange, BM = Brown, PEL = Blue). In the Genetic Differentiation column the number of front comparisons is given for each taxon, with the inner circle representing the frequency of comparisons showing genetic reduction patterns (YES = dark grey, NO = light grey), and the outer circle representing the frequency of significant genetic differences between localities separated by a front (YES = dark grey, NO = light grey).

Life history traits such as the length of PLD or the adult habitat show similar patterns across different taxa that seem to relate with genetic differentiation independent of phylogenetic relationships (Fig 6). For instance, species with low dispersal capabilities, in all taxa, frequently present significant differentiation between populations separated by fronts but not related to the front reducing connectivity (Fig 6).

For each dataset we also compared with a Wilcoxon matched pairs test the standardized F_ST_ distances between localities separated by the front (F) and those without front (NF). No significant differences were observed for each front analysed separately or all together, despite having more species with higher standardized differentiation between populations when separated by a front (Table 2). The effect of different factors and their interactions (FRONT, PLD, LIFE and MARKER) on gene flow reduction was assessed with PERMANOVA using the standardized NDIF as dependent continuous variable. Only PLD showed a significant effect (Table 3). The differentiation between localities was not dependent on the front assayed nor the marker used (Table 3). Unfortunately, the variable LIFE_PLD combined could not be compared for each front since increasing the number of categories drastically reduces the number of comparisons to be assessed. More population genetic studies in a wider number of species with different dispersal capabilities are needed to statistically analyse this variable.

**Table 2:** Wilcoxon matched pairs test for each oceanographic front comparing the standardized F_ST_ values between localities separated by the front (F) and those without front (NF).

Front	n	NF>F	NF<F	Z	p
GS	24	8	16	1.06	0.290
AOF	28	12	16	1.02	0.305
IC	36	17	19	0.16	0.875
BF	23	9	14	0.73	0.465
SC	28	14	14	0.48	0.631
ADR	17	6	11	0.17	0.868
AEG	20	7	13	1.08	0.279
Total	176	73	103	1.57	0.117

NF>F indicates that gene flow is larger between localities not separated by a front. NF<F indicates the contrary. The fronts are GS (Gibraltar Strait), AOF (Almeria-Oran Front), IC (Ibiza Channel), BF (Balearic Front), SC (Sicily Channel), ADR (Adriatic Sea), AEG (Aegean Sea). n = number of species compared in each front. Total combines all comparisons including the possibility of multiple fronts for the same species.

**Table 3:** PERMANOVA analyses considering the effect of different variables and their interaction on gene flow reduction.

	df	Pseudo-F	p (perms)
FRONT	6	0.64	0.67
PLD	2	8.47	**0.00**
FRONT × PLD	12	0.57	0.86
FRONT	6	0.11	0.99
LIFE	2	2.28	0.12
FRONT × LIFE	12	0.80	0.57
FRONT	6	0.34	0.91
MARKER	1	0.79	0.38
FRONT × MARKER	6	0.49	0.82

Significant p values in bold.

## Discussion

**Fig 7:**
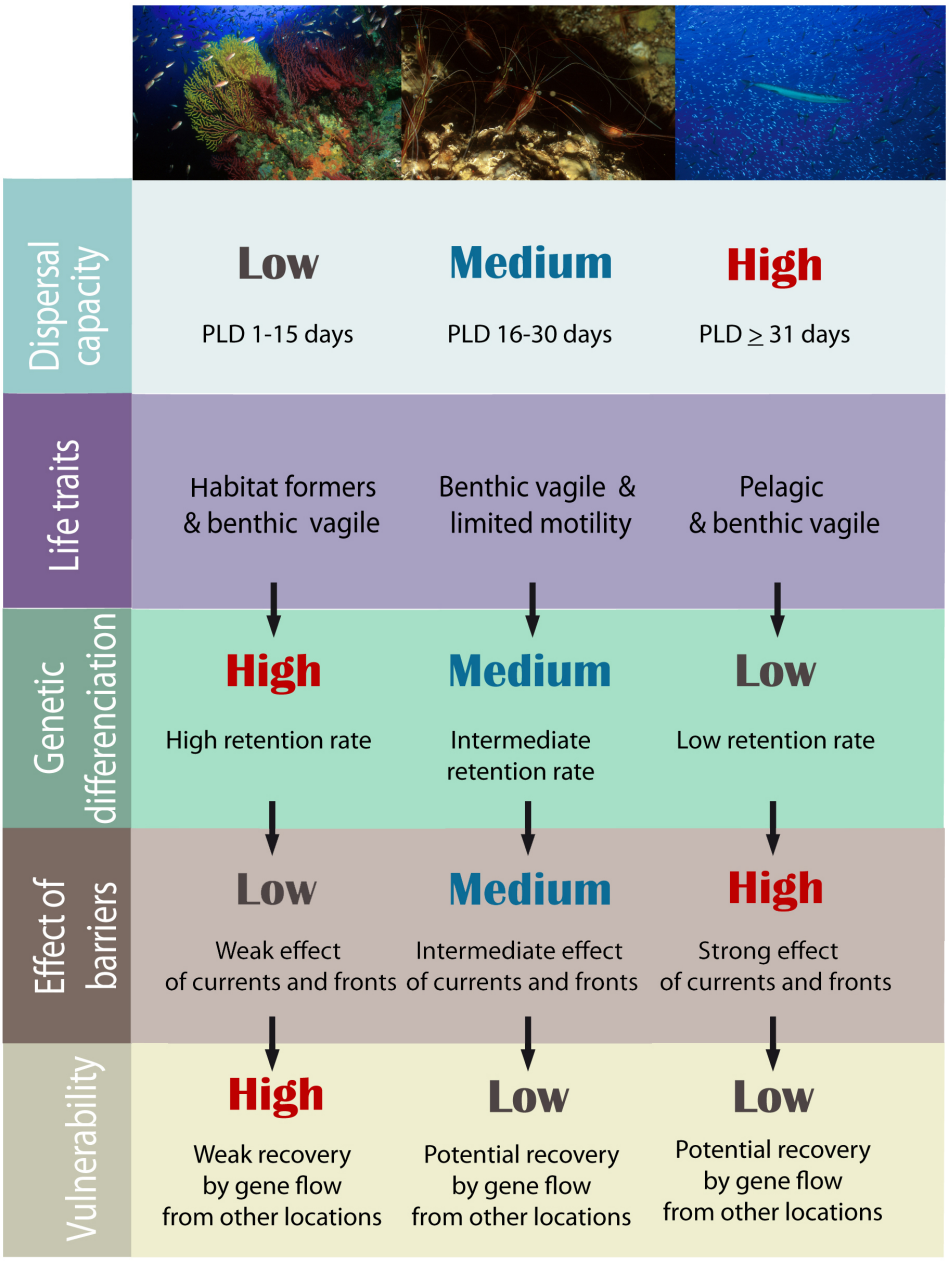
Effect of life history traits on population genetic differentiation and their impact on connectivity reduction mediated by oceanographic discontinuities. These effects influence the capacity of population recovery and species vulnerability.

The results of the present study demonstrate that the oceanographic discontinuities in the Mediterranean Sea, in general, affect gene flow among localities. However, this effect is not homogeneous considering the life history trait of species (Fig 7), not restricted to phylogenetic groups. Species with short dispersal capabilities showed significant genetic differentiation between most locations, but the gene flow reduction was not mediated by the presence of a front. However, significant connectivity reduction due to an oceanographic front was detected in species with PLD longer than 2–4 weeks and adult mobility. The variability in this effect can have important consequences in the analysis of the effective connectivity processes in the Mediterranean Sea [32,33] and in the identification of natural conservation units for the establishment of an MPA network or in conservation strategies [8,34].

### Life history traits and gene flow reduction

It is now more widely accepted that marine species can have strong genetic population structuring even on very small scales dismissing the previous hypothesis of open, homogeneous and interconnected seas. This strong population structure can be caused by oceanographic barriers such as the Almeria-Oran Front [21,27,61], or life history traits such as larval behaviour [62] and Isolation By Distance [63]. We show that genetic differences among localities are highly dependent on life history traits of species. The highest significant differences occur in sessile benthic species with short planktonic larval durations (PLD < two weeks) independently of the presence of a front.

PLD has a strong influence on the genetic structure but also on the geographical distribution of species [64] with adult traits being determinant as well [65]. The larvae of species with a short PLD in the Mediterranean Sea usually remain close to the coastline [57,66] and disperse only a few meters/kilometres from the parents, as seen for example in sponges or gorgonians [62,67]. Therefore, the population differentiation in these species seems mostly caused by the reduced dispersal capabilities of the larvae since their adults are sessile. For the sponge *Crambe crambe* the estimated mean dispersal distances per generation were only about 35 cm, suggesting that the observed fine-scale genetic structure may be common in invertebrates with lecitotrophic larvae [14]. A similar result was observed in the coral *Astroides calycularis* [68], where the low connectivity is explained by the negative buoyancy and demersal behaviour of the larvae. In the gorgonian *Paramuricea clavata* high levels of self-recruitment and parentage relationships were detected at a small scale [69] with genetic drift having a strong impact in populations [70]. No isolation by distance and high genetic differentiation between localities separated by dozens to hundreds of kilometres was detected in the seagrass *Zostera noltei*, where genetic and physical connectivity assessment also indicated that rare long distance dispersal was possible [71]. These characteristics can generate strong genetic differentiation among geographically closed localities and similarities between widely separated localities, independently of the presence or absence of an oceanographic discontinuity.

For most species with longer PLDs (> two weeks) larvae move along the continental shelf and slope [66]. The distribution of their larvae are strongly affected by currents [72,73], eddies [74] and oceanographic fronts [75]. In these species, genetic differentiation between control locations (no-front, NF) was generally smaller than between localities separated by oceanographic discontinuities. This difference indicates that the front has an additional effect reducing gene flow among localities, although genetic differentiation between localities was seldom significant. Therefore, most fish and crustacean species living on the shelf and slope (e.g. *Diplodus vulgaris*, *Mullus* spp., *Serranus cabrilla*, *Liocarcinus depurator*) present a genetic structure generated by the presence of fronts [17,21,25,28]. However, as several authors have pointed out, limited larval dispersal can also be observed in species with long PLD (>2 weeks) [6,76,77]. Habitat suitability may have important implication; in the fish *Tripterygion delaisi* the presence of continuous rocky habitat between localities prevents genetic differentiation, while large discontinuities of sand or deep-water channels seem to reduce gene flow [78]. The largest distance between recruitment locations in this species measured through kinship analysis was ca. 11.5 km [79]. These direct measurements on population connectivity can provide compelling evidence to estimate the size and distance among areas in order to link them into an ecologically coherent MPAs network.

### Gene flow reduction by oceanographic front

The oceanographic discontinuities in the Mediterranean Sea restrict gene flow in numerous species. However, the dynamic behaviour of these fronts shows significant intra- and inter-annual variability [80,81]. When all fronts were analysed simultaneously, we observed gene flow reduction in 58.8% of the comparisons. This tendency seems to apply to all fronts with the smallest reduction observed in the Sicily Channel (SC) and the largest in the Gibraltar Strait (GS). Differences between fronts could be due to the species studied and to fluctuations in the front strength. The AOF has been described as the main oceanographic barrier causing genetic differentiation along the Atlantic-Mediterranean transition area [27,82]. However, this front presents inter-annual variability allowing some degree of homogenization between localities of the same species at certain years [22]. Therefore, although most fronts caused a clear restriction in gene flow, the effect of oceanographic barriers might be temporarily relaxed and different results obtained regarding the year of dispersal of the analysed samples [22,83]. The reproductive period of species may also determine whether fronts are the cause of the genetic structuring. Across the Ibiza Channel, localities will be genetically differentiated if the reproduction coincides with the largest intensity of the front, as shown for *Serranus cabrilla* [17], and not differentiated if the reproduction period coincides with the lowest intensity of the front, as observed in *Epinephelus marginatus* [84].

### Potential effect in the establishment of a MPA network and stock identifications in the Mediterranean Sea

The identification of population units and the definition of population boundaries are one of the highest priorities in the management of marine ecosystems [85,86]. Studies on the connectivity assessment between MPA networks, with an integrated multispecies approach encompassing oceanography, dispersal capabilities and genetics, are strongly needed [34,87]. In order to implement functional networks of conservation a number of umbrella species representatives of the ecosystem should be selected and analysed with genetic tools [88]. Therefore, we recommend that species with different life histories to be selected within the umbrella species group as they provide different, but complementary information on connectivity of the whole ecosystem. The role of oceanographic fronts in the restriction of gene flow in numerous species supports the eco-regionalization of the Mediterranean Sea proposed by different studies [32,33]. This regionalization based on dispersal patterns and connectivity mostly affect species with longer PLDs, a common characteristic of most commercial species (e.g. fishes and crustaceans), and any protection measures, including the establishment of MPAs networks or management plans, should consider them.

However, the establishment of MPA networks must contemplate the whole ecosystem, with special caution to those species with low dispersal capabilities. Oceanographic discontinuities did not generally affect the connectivity patterns in species with low dispersal larval capabilities (PLD < 2 weeks). These species include numerous sponges, gorgonians, angiosperms that are habitat formers [47,89] and essential in providing refuges or food for settlers of fishes [90]. The so-called “ecosystem engineers”, i.e. species that generate new habitat for other species, need an adequate protection since their elimination will be hard to recover given their low dispersal potential capabilities [91]. In the Mediterranean Sea destructive fishing practices, eutrophication and coastal development are among the major impacts responsible for habitat change [92] and in numerous ecosystems the “engineers species” have disappeared [93,94]. Furthermore, reducing cumulative local human impacts cannot reverse the loss of natural capital and the recovery of these species only occurs by transplanting propagules or juvenile stages [95] since most algae, sponges and other “engineers species” disperse only few meters and, in general, less than 1 km [91]. Moreover, local adaptation processes should also be considered in restoration actions given the importance of genetic adaptation to environmental pressures [96,97].

An optimal MPA network should protect areas of adequate size, including different home ranges, to help engineer species survive and flourish, thus providing habitat and food for larger species such as commercial fish species. Our results confirm the study of Shanks et al.[18] that suggested for marine reserve networks to be designed large enough (ca. 4–6 km in diameter) to contain short-distance dispersing larvae and be spaced far enough (ca. 10–20 km) for long-distance dispersing propagules released from one reserve to be able to settle in adjacent reserves [8]. These protected areas should be further interconnected providing suitable settlement habitat for far travelling larvae [98]. Finally, these networks need be created on either side of oceanographic fronts, since they generally are barriers to gene flow and often determine boundaries between hydrodynamic provinces [33], ensuring resilience of the whole ecosystem.

## Conclusions

Genetic connectivity is important for recovery from anthropogenic of natural impacts. We show that both larval and adult life characteristics impact connectivity among localities and across oceanographic discontinuities. We observed that species with lower mobility potential have higher frequency of pairwise localities showing significant genetic differentiation than species with higher motility capacity, but independent of the existence of fronts and more subject to genetic drift. Moreover, oceanographic discontinuities reduce gene flow in species with medium to high dispersal abilities (Fig 7). We encourage, for the resilience of the whole ecosystem, to consider oceanographic discontinuities and umbrella species with different life characteristics when identifying management units and for designing networks of Marine Protected Areas.

## Supporting information

S1 Checklist PRISMA Checklist for current study. (PDF)

S1 Table List of studies and species considered in the present study for the seven Mediterranean oceanographic discontinuities showing significant genetic distances and gene flow reduction by oceanographic front. (PDF)

S2 Table Genetic and geographic distances between localities separated by fronts and no-fronts for the 177 datasets. (PDF)

S3 Table Number of species, for each PLD category and LIFE strategy, showing Non significant/Significant genetic distances and No reduction/ Reduction gene flow between localities separated by each oceanographic front. (PDF)
