# Supplementary material for: Impact of life history traits on gene flow: A multispecies systematic review across oceanographic barriers in the Mediterranean Sea
Source: PLoS One. 2017 May 10;12(5):e0176419. doi: 10.1371/journal.pone.0176419 (PMC5425013; doi:10.1371/journal.pone.0176419)
Supplement: S1 Table — (PDF) [file pone.0176419.s002.pdf]

Supporting information

S1 Table. **List of studies and species considered in the present study for the seven Mediterranean oceanographic discontinuities showing significant genetic distances and gene flow reduction by oceanographic front.** Species considered in the present study identified according to taxonomic group, LIFE strategy (LIFE: BS= Benthic sessile or low motility, BM= benthic vagile, PEL= pelagic), Planktonic Larval Duration (PLD: L≥31 days, M=16-30 days, S=1-15 days), their combination (LIFE_PLD) and Marker used (MT= COI, 16S, cyt b, NUC=allozymes, microsatellites, SNPs). Genetic significance indicates the existence of genetic differentiation (1) or no genetic differentiation (0) for each population pairwise comparison as stated in each study. We considered connectivity reduction mediated by the front (1) when the standardized F_{ST} by geographic distance between localities separated by a front was larger than between control localities with no-front (NF), otherwise genetic differences could not be associated to the front effect (0). The fronts analysed are: Gibraltar Strait (GS), Almeria-Oran Front (AOF), Ibiza Channel (IC), Balearic Front (BF), Sicily Channel (SC), Otranto Channel (ADR), and the southern margin of the Aegean Sea (AEG).

**Taxonomic group**	**Species**	**References**	**LIFE**	**PLD**	**LIFE_PLD**	**Marker**	**Genetic significance / Connectivity reduction**							
							**NF**	**GS**	**AOF**	**IC**	**BF**	**SC**	**ADR**	**AEG**
Angiosperm	*Cymodocea nodosa*	[1]	BS	S	BSS	NUC	1	1/0	1/0	1/0	1/0		1/0	1/0
Porifera	*Crambe crambe*	[2]	BS	S	BSS	MT	1			0/0	0/0			
Porifera	*Ircinia fasciculata*	[3]	BS	S	BSS	NUC	0		0/1	0/0	0/1			
Porifera	*Paraleucilla magna*	[4]	BS	S	BSS	NUC	0	1/0	1/1	1/0				
Porifera	*Scopalina lophyropoda*	[5]	BS	S	BSS	NUC	1			1/1				
Porifera	*Spongia lamella*	[6]	BS	S	BSS	NUC	1				1/1			

**Group**	**Species**	**References**	**LIFE**	**PLD**	**LIFE_PLD**	**Marker**	**NF**	**GS**	**AOF**	**IC**	**BF**	**SC**	**ADR**	**AEG**
Porifera	*Spongia officinalis*	[7]	BS	S	BSS	NUC	1							1/1
Cnidaria	*Astroides calycularis*	[8]	BS	S	BSS	NUC	1		1/1					
Cnidaria	*Aurelia aurita*	[9]	PEL	L	PELL	NUC	0					1/1		
Cnidaria	*Cladocora caespitosa*	[10]	BS	S	BSS	NUC	1			1/0	1/0			
Cnidaria	*Corallium rubrum*	[11]	BS	S	BSS	NUC	1				1/0			
Cnidaria	*Paramuricea clavata*	[12]	BS	S	BSS	NUC	1		1/0	1/1				
Mollusca	*Donax trunculus*	[13]	BS	M	BSM	NUC	0	1/1						
Mollusca	*Haliotis tuberculata*	[14]	BS	S	BSS	NUC	0			0/0	0/1			
Mollusca	*Mytilus galloprovincialis*	[15]	BS	M	BSM	NUC	1	1/0	1/0	0/0				
Mollusca	*Octopus vulgaris*	[16]	BM	S	BMS	MT	1						0/0	
Mollusca	*Octopus vulgaris*	[17]	BM	S	BMS	MT	1					1/0		
Mollusca	*Patella rustica*	[18]	BS	M	BSM	NUC	0	1/1						
Mollusca	*Patella rustica*	[18]	BS	M	BSM	MT	0						1/0	
Mollusca	*Patella ulyssiponensis*	[19]	BS	M	BSM	MT	0	1/1				0/0		
Mollusca	*Pinna nobilis*	[20]	BS	S	BSS	MT	0					0/1	0/1	1/1
Mollusca	*Ruditapes decussatus*	[21]	BS	M	BSM	MT	0					0/0		
Mollusca	*Sepia officinalis*	[22]	BM	S	BMS	NUC	0	1/1	1/1	1/1				
Mollusca	*Sepia officinalis*	[23]	BM	S	BMS	MT	0				1/1	0/1	0/1	1/1
Mollusca	*Solen marginatus*	[24]	BS	M	BSM	NUC	0					1/0		
Crustacea	*Aristeomorpha foliacea*	[25]	BM	L	BML	NUC	0							0/0
Crustacea	*Carcinus aestuarii*	[26]	BM	M	BMM	NUC	0					1/1	1/1	
Crustacea	*Liocarcinus depurator*	[27]	BM	M	BMM	MT	0	1/1	0/0	1/1				
Crustacea	*Macropipus tuberculatus*	[27]	BM	M	BMM	MT	0	0/1	0/1	0/1				
Crustacea	*Munida intermedia*	[27]	BM	M	BMM	MT	0	0/1	0/0	0/0				
Crustacea	*Pachygrapsus marmoratus*	[28]	BM	L	BML	MT	0	0/0				0/0	0/0	0/0

**Group**	**Species**	**References**	**LIFE**	**PLD**	**LIFE_PLD**	**Marker**	**NF**	**GS**	**AOF**	**IC**	**BF**	**SC**	**ADR**	**AEG**
Crustacea	*Pagurus alatus*	[27]	BM	M	BMM	MT	0	0/1	0/1	0/1				
Crustacea	*Pagurus excavatus*	[27]	BM	M	BMM	MT	0	0/1	0/0	0/1				
Crustacea	*Palaemon elegans*	[29]	BM	L	BML	MT	0	0/1				0/0		
Crustacea	*Palinurus elephas*	[30]	BM	L	BML	NUC	0				1/1			
Crustacea	*Parapenaeus longirostris*	[27]	BM	L	BML	MT	0	0/1	0/0	0/1				
Crustacea	*Parapenaeus longirostris*	[31]	BM	L	BML	MT	0					0/0	0/0	1/1
Crustacea	*Penaeus kerathurus*	[32]	BM	M	BMM	MT	0					1/1		
Crustacea	*Plesionika heterocarpus*	[27]	BM	L	BML	MT	0	0/1	0/1	0/1				
Crustacea	*Stenosoma nadejda*	[33]	BM	S	BMS	MT	1	1/0	1/0					
Echinodermata	*Arbacia lixula*	[34]	BS	M	BSM	MT	0		0/1	0/1	0/0			
Echinodermata	*Astropecten aranciacus*	[35]	BS	L	BSL	NUC	0	0/1						
Echinodermata	*Echinaster sepisotus*	[36]	BM	L	BML	NUC	0			0/1	0/1	0/1	0/1	
Echinodermata	*Holothuria mammata*	[37]	BS	L	BSL	MT	0			0/1				
Echinodermata	*Holothuria polii*	[38]	BS	L	BSL	NUC	1					1/0		
Echinodermata	*Holothuria polii*	[39]	BS	L	BSL	MT	0			0/0	0/1			
Echinodermata	*Paracentrotus lividus*	[40]	BS	M	BSM	MT	0				0/1			
Echinodermata	*Paracentrotus lividus*	[41]	BS	M	BSM	MT	0	0/0	0/1	0/1				
Echinodermata	*Parastichopus regalis*	[42]	BS	L	BSL	MT	0		1/1	0/1				
Tunicata	*Pseudodistoma crucigaster*	[43]	BS	S	BSS	MT	1				1/0			
Pisces	*Anguilla anguilla*	[44]	BM	L	BML	NUC	0	0/0						
Pisces	*Apogon imberbis*	[45]	BM	M	BMM	NUC	0		1/0	0/0	1/1			
Pisces	*Atherina boyeri*	[46]	BM	S	BMS	NUC	1						1/0	
Pisces	*Chromis chromis*	[47]	BM	M	BMM	MT	0					0/1		1/1
Pisces	*Coris julis*	[48]	BM	L	BML	MT	0				0/0	0/1	0/1	0/1
Pisces	*Coryphoblennius galerita*	[49]	BM	M	BMM	MT	1			0/0				

**Group**	**Species**	**References**	**LIFE**	**PLD**	**LIFE_PLD**	**Marker**	**NF**	**GS**	**AOF**	**IC**	**BF**	**SC**	**ADR**	**AEG**
Pisces	*Dicentrarchus labrax*	[50]	PEL	L	PELL	NUC	0					0/1	0/1	1/1
Pisces	*Dicentrarchus labrax*	[51]	PEL	L	PELL	NUC	0	1/1	1/1	1/1				
Pisces	*Diplodus sargus*	[52]	BM	M	BMM	NUC	1					1/0		
Pisces	*Diplodus vulgaris*	[45]	BM	L	BML	NUC	1		1/1	1/1	0/1			
Pisces	*Engraulis encrasicolus*	[53]	PEL	L	PELL	NUC	0	0/1				0/0	0/1	0/1
Pisces	*Epinephelus marginatus*	[54]	BM	M	BMM	NUC	0			0/0	0/0	1/0		0/0
Pisces	*Merluccius merluccius*	[55]	BM	L	BML	NUC	0	1/1				0/1	0/1	0/1
Pisces	*Mugil cephalus*	[56]	PEL	L	PELL	NUC	0			0/1		0/0		0/1
Pisces	*Mullus barbatus*	[57]	BM	L	BML	NUC	1			1/0				
Pisces	*Mullus barbatus*	[58]	BM	L	BML	NUC	0							0/0
Pisces	*Mullus barbatus*	[59]	BM	L	BML	NUC	0					1/0		
Pisces	*Mullus barbatus*	[60]	BM	L	BML	NUC	0		0/0					
Pisces	*Mullus surmuletus*	[45]	BM	L	BML	NUC	1		1/0	0/0	1/1			
Pisces	*Oblada melanura*	[45]	PEL	M	PELM	NUC	1		0/0	1/0	1/1			
Pisces	*Sardina pilchardus*	[61]	PEL	L	PELL	NUC	0	1/1						
Pisces	*Sardina pilchardus*	[62]	PEL	L	PELL	NUC	1		1/1	1/1				
Pisces	*Sardina pilchardus*	[63]	PEL	L	PELL	NUC	0						0/1	
Pisces	*Scorpaena porcus*	[64]	BM	M	BMM	NUC	0						0/1	
Pisces	*Scyliorhinus canicula*	[65]	BM	S	BMS	NUC	0							1/1
Pisces	*Seriola dumerilli*	[66]	PEL	L	PELL	MT	0					0/1		
Pisces	*Serranus cabrilla*	[67]	BM	M	BMM	NUC	0		1/1	1/1	0/1	0/1		0/1
Pisces	*Solea vulgaris*	[68]	BM	M	BMM	MT	0					1/1	0/1	
Pisces	*Solea vulgaris*	[69]	BM	M	BMM	NUC	0							0/0
Pisces	*Squalus blainville*	[70]	BM	S	BMS	MT	0					0/1		0/1
Pisces	*Symphodus tinca*	[45]	BM	S	BMS	NUC	1		1/1	0/0	0/1			

Group	Species	References	LIFE	PLD	LIFE_PLD	Marker	NF	GS	AOF	IC	BF	SC	ADR	AEG
Pisces	*Thalassoma pavo*	[71]	BM	L	BML	MT	0					0/0		0/1
Pisces	*Tripterigion delaisi*	[72]	BM	M	BMM	NUC	1		1/1	1/0	1/0			

References

1. Alberto F, Massa S, Manent P, Diaz-Almela E, Arnaud-Haond S, Duarte CM, et al. Genetic differentiation and secondary contact zone in the seagrass *Cymodocea nodosa* across the Mediterranean-Atlantic transition region. J Biogeogr. 2008;35: 1279–1294. doi:10.1111/j.1365-2699.2007.01876.x

2. Duran S, Pascual M, Turon X. Low levels of genetic variation in mtDNA sequences over the western Mediterranean and Atlantic range of the sponge *Crambe crambe* (Poecilosclerida). Mar Biol. 2004;144: 31–35. doi:10.1007/s00227-003-1178-5

3. Riesgo A, Pérez-Portela R, Pita L, Blasco G, Erwin PM, López-Legentil S. Population structure and connectivity in the Mediterranean sponge Ircinia fasciculata are affected by mass mortalities and hybridization. Heredity (Edinb). Nature Publishing Group; 2016;117: 1–13. doi:10.1038/hdy.2016.41

4. Guardiola M, Frotscher J, Uriz MJ. High genetic diversity, phenotypic plasticity, and invasive potential of a recently introduced calcareous sponge, fast spreading across the Atlanto-Mediterranean basin. Mar Biol. Springer Berlin Heidelberg; 2016;163: 123. doi:10.1007/s00227-016-2862-6

5. Blanquer A, Uriz MJ. Population genetics at three spatial scales of a rare sponge living in fragmented habitats. Bmc Evol Biol. 2010;10. doi:1310.1186/1471-2148-10-13

6. Pérez-Portela R, Noyer C, Becerro MA. Genetic structure and diversity of the endangered bath sponge *Spongia lamella*. Aquat Conserv Mar Freshw Ecosyst. 2015;25: 365–379. doi:10.1002/aqc.2423

7. Dailianis T, Tsigenopoulos CS, Dounas C, Voultsiadou E. Genetic diversity of the imperilled bath sponge *Spongia officinalis* Linnaeus, 1759 across the Mediterranean Sea: Patterns of population differentiation and implications for taxonomy and conservation. Mol Ecol. Blackwell Publishing Ltd; 2011;20: 3757–3772. doi:10.1111/j.1365-294X.2011.05222.x

8. Casado-Amezúa P, Goffredo S, Templado J, Machordom A. Genetic assessment of population structure and connectivity in the threatened mediterranean coral *Astroides calycularis* (Scleractinia, Dendrophylliidae) at different spatial scales. Mol Ecol. 2012;21: 3671–3685. doi:10.1111/j.1365-294X.2012.05655.x

9. Ben Faleh A, Ben Othmen A, Deli T, Annabi A, Said K. High genetic homogeneity of the moon jelly *Aurelia aurita* (Scyphozoa, Semaeostomeae) along the Mediterranean coast of Tunisia. African J Mar Sci. Taylor & Francis Group; 2009;31: 73–80. doi:10.2989/AJMS.2009.31.1.6.777

10. Casado-Amezúa P, Kersting DK, Templado J, Machordom A. Regional genetic differentiation among populations of *Cladocora caespitosa* in the Western Mediterranean. Coral Reefs. Springer Berlin Heidelberg; 2014;33: 1031–1040. doi:10.1007/s00338-014-1195-5

11. Ledoux JB, Mokhtar-Jamai K, Roby C, Feral JP, Garrabou J, Aurelle D. Genetic survey of shallow populations of the Mediterranean red coral *Corallium rubrum*

(Linnaeus, 1758) : new insights into evolutionary processes shaping nuclear diversity and implications for conservation. Mol Ecol. 2010;19: 675–690. doi:10.1111/j.1365-294X.2009.04516.x

12. Mokhtar-Jamaï K, Pascual M, Ledoux JB, Coma R, Féral JP, Garrabou J, et al. From global to local genetic structuring in the red gorgonian *Paramuricea clavata*: The interplay between oceanographic conditions and limited larval dispersal. Mol Ecol. 2011;20: 3291–3305. doi:10.1111/j.1365-294X.2011.05176.x

13. Marie AD, Lejeusne C, Karapatsiou E, Cuesta JA, Drake P, Macpherson E, et al. Implications for management and conservation of the population genetic structure of the wedge clam Donax trunculus across two biogeographic boundaries. Sci Rep. 2016;6. doi:10.1038/srep39152

14. Roussel V, van Wormhoudt A. The Effect of Pleistocene Climate Fluctuations on Distribution of European Abalone (*Haliotis tuberculata*), Revealed by Combined Mitochondrial and Nuclear Marker Analyses. Biochemical Genetics. Springer US; 20 Oct 2016: 1–31. doi:10.1007/s10528-016-9778-1

15. Diz AP, Presa P. Regional patterns of microsatellite variation in Mytilus galloprovincialis from the Iberian Peninsula. Mar Biol. 2008;154: 277–286. doi:10.1007/s00227-008-0921-3

16. De Luca D, Catanese G, Procaccini G, Fiorito G. *Octopus vulgaris* (Cuvier, 1797) in the Mediterranean Sea: Genetic diversity and population structure. Zane L, editor. PLoS One. Public Library of Science; 2016;11: e0149496. doi:10.1371/journal.pone.0149496

17. Fadhlaoui-Zid K, Knittweis L, Aurelle D, Nafkha C, Ezzeddine S, Fiorentino F, et al. Genetic structure of *Octopus vulgaris* (Cephalopoda, Octopodidae) in the central Mediterranean Sea inferred from the mitochondrial COIII gene. Comptes Rendus - Biol. 2012;335: 625–636. doi:10.1016/j.crvi.2012.10.004

18. Sá-Pinto A, Branco MS, Alexandrino PB, Fontaine MC, Baird SJE. Barriers to Gene Flow in the Marine Environment: Insights from Two Common Intertidal Limpet Species of the Atlantic and Mediterranean. Steinke D, editor. PLoS One. Public Library of Science; 2012;7: e50330. doi:10.1371/journal.pone.0050330

19. Cossu P, Dedola GL, Scarpa F, Sanna D, Lai T, Maltagliati F, et al. Patterns of spatial genetic variation in *Patella ulyssiponensis*: insights from the western Mediterranean marine ecoregion. Hydrobiologia. 2015;755: 39–55. doi:10.1007/s10750-015-2216-2

20. Sanna D, Cossu P, Dedola GL, Scarpa F, Maltagliati F, Castelli A, et al. Mitochondrial DNA Reveals Genetic Structuring of *Pinna nobilis* across the Mediterranean Sea. Colgan DJ, editor. PLoS One. Public Library of Science; 2013;8: e67372. doi:10.1371/journal.pone.0067372

21. Gharbi A, Chatti N, Said K, Wormhoudt A. Genetic variation and population structure of the carpet shell clam *Ruditapes decussatus* along the Tunisian coast inferred from mtDNA and ITS1 sequence analysis. Biologia (Bratisl). SP Versita; 2010;65: 688–696. doi:10.2478/s11756-010-0069-8

22. Pérez-Losada M, Guerra A, Carvalho GR, Sanjuan A, Shaw PW. Extensive population subdivision of the cuttlefish *Sepia officinalis* (Mollusca : Cephalopoda) around the Iberian Peninsula indicated by microsatellite DNA variation. Heredity (Edinb). 2002;89: 417–424. doi:10.1038/sj.hdy.6800160

23. Pérez-Losada M, Nolte MJ, Crandall KA, Shaw PW. Testing hypotheses of population structuring in the Northeast Atlantic Ocean and Mediterranean Sea using the common cuttlefish *Sepia officinalis*. Mol Ecol. 2007;16: 2667–2679. doi:10.1111/j.1365-294X.2007.03333.x

24. Hmida L, Fassatoui C, Ayed D, Ayache N, Romdhane MS. Genetic characterization of the razor clam *Solen marginatus* (Mollusca: Bivalvia: Solenidae) in Tunisian coasts based on isozyme markers. Biochem Syst Ecol. 2012;40: 146–155. doi:10.1016/j.bse.2011.10.016

25. Fernández MV, Maltagliati F, Pannacciulli FG, Roldán MI. Analysis of genetic variability in *Aristaeomorpha foliacea* (Crustacea, Aristeidae) using DNA-ISSR (Inter Simple Sequence Repeats) markers. Comptes Rendus - Biol. 2011;334: 705–712. doi:10.1016/j.crvi.2011.07.005

26. Schiavina M, Marino IAM, Zane L, Melià P. Matching oceanography and genetics at the basin scale. Seascape connectivity of the Mediterranean shore crab in the Adriatic Sea. Mol Ecol. 2014;23: 5496–5507. doi:10.1111/mec.12956

27. García-Merchán VH, Robainas-Barcia A, Abelló P, Macpherson E, Palero F, García-Rodríguez M, et al. Phylogeographic patterns of decapod crustaceans at the Atlantic–Mediterranean transition. Mol Phylogenet Evol. 2012;62: 664–672. doi:10.1016/j.ympev.2011.11.009

28. Fratini S, Ragionieri L, Deli T, Harrer A, Marino IAM, Cannicci S, et al. Unravelling population genetic structure with mitochondrial DNA in a notional panmictic coastal crab species: sample size makes the difference. BMC Evol Biol. 2016;16: 150. doi:10.1186/s12862-016-0720-2

29. Reuschel S, Cuesta JA, Schubart CD. Marine biogeographic boundaries and human introduction along the European coast revealed by phylogeography of the prawn *Palaemon elegans*. Mol Phylogenet Evol. 2010;55: 765–775. doi:10.1016/j.ympev.2010.03.021

30. Babbucci M, Buccoli S, Cau A, Cannas R, Goñi R, Díaz D, et al. Population structure, demographic history, and selective processes: Contrasting evidences from mitochondrial and nuclear markers in the European spiny lobster *Palinurus elephas* (Fabricius, 1787). Mol Phylogenet Evol. 2010;56: 1040–1050. doi:10.1016/j.ympev.2010.05.014

31. Lo Brutto S, Maggio T, Arculeo M. Isolation By Distance (IBD) signals in the deep-water rose shrimp *Parapenaeus longirostris* (Lucas, 1846) (Decapoda, Panaeidae) in the Mediterranean Sea. Mar Environ Res. 2013;90: 1–8. doi:10.1016/j.marenvres.2013.05.006

32. Zitari-Chatti R, Chatti N, Fulgione D, Caiazza I, Aprea G, Elouaer A, et al. Mitochondrial DNA variation in the caramote prawn *Penaeus (Melicertus) kerathurus* across a transition zone in the Mediterranean Sea. Genetica.

2009;136: 439–447. doi:10.1007/s10709-008-9344-9

33. Xavier R, Zenboudji S, Lima FP, Harris DJ, Santos AM, Branco M. Phylogeography of the marine isopod *Stenosoma nadejda* (Rezig, 1989) in North African Atlantic and western Mediterranean coasts reveals complex differentiation patterns and a new species. Biol J Linn Soc. Blackwell Publishing Ltd; 2011;104: 419–431. doi:10.1111/j.1095-8312.2011.01718.x

34. Wangensteen OS, Turon X, Pérez-Portela R, Palacín C. Natural or Naturalized? Phylogeography Suggests That the Abundant Sea Urchin *Arbacia lixula* Is a Recent Colonizer of the Mediterranean. Dupont S, editor. PLoS One. Public Library of Science; 2012;7: e45067. doi:10.1371/journal.pone.0045067

35. Zulliger DE, Tanner S, Ruch M, Ribi G. Genetic structure of the high dispersal Atlanto-Mediterreanean sea star *Astropecten aranciacus* revealed by mitochondrial DNA sequences and microsatellite loci. Mar Biol. 2009;156: 597–610. doi:10.1007/s00227-008-1111-z

36. Garcia-Cisneros A, Palacín C, Ben Khadra Y, Pérez-Portela R. Low genetic diversity and recent demographic expansion in the red starfish *Echinaster sepositus* (Retzius 1816). Sci Rep. Nature Publishing Group; 2016;6: 33269. doi:10.1038/srep33269

37. Borrero-Pérez GH, González-Wangüemert M, Marcos C, Pérez-Ruzafa A. Phylogeography of the Atlanto-Mediterranean sea cucumber *Holothuria (Holothuria) mammata*: The combined effects of historical processes and current oceanographical pattern. Mol Ecol. 2011;20: 1964–1975. doi:10.1111/j.1365-294X.2011.05068.x

38. Gharbi A, Said K. Genetic variation and population structure of *Holothuria polii* from the eastern and western Mediterranean coasts in Tunisia. J Mar Biol Assoc United Kingdom. Cambridge University Press; 2011;91: 1599–1606. doi:10.1017/S0025315411000245

39. Valente S, Serrão EA, González-Wangüemert M. West versus East Mediterranean Sea: origin and genetic differentiation of the sea cucumber *Holothuria polii*. Mar Ecol. 2015;36: 485–495. doi:10.1111/maec.12156

40. Calderón I, Giribet G, Turon X. Two markers and one history: phylogeography of the edible common sea urchin *Paracentrotus lividus* in the Lusitanian region. Mar Biol. 2008;154: 137–151. doi:10.1007/s00227-008-0908-0

41. Calderón I, Pita L, Brusciotti S, Palacín C, Turon X. Time and space: genetic structure of the cohorts of the common sea urchin *Paracentrotus lividus* in Western Mediterranean. Mar Biol. 2012;159: 187–197. doi:10.1007/s00227-011-1799-z

42. Maggi C, González-Wangüemert M. Genetic differentiation among *Parastichopus regalis* populations in the Western Mediterranean Sea: Potential effects from its fishery and current connectivity. Mediterr Mar Sci. 2015;16: 489–501. doi:10.12681/mms.1020

43. Tarjuelo I, Posada D, Crandall KA, Pascual M, Turon X. Phylogeography and

speciation of colour morphs in the colonial ascidian *Pseudodistoma crucigaster*. Mol Ecol. 2004;13: 3125–3136. doi:10.1111/j.1365-294X.2004.02306.x

44. Als TD, Hansen MM, Maes GE, Castonguay M, Riemann L, Aarestrup K, et al. All roads lead to home: Panmixia of European eel in the Sargasso Sea. Mol Ecol. 2011;20: 1333–1346. doi:10.1111/j.1365-294X.2011.05011.x

45. Galarza JA, Carreras-Carbonell J, Macpherson E, Pascual M, Roques S, Turner GF, et al. The influence of oceanographic fronts and early-life-history traits on connectivity among littoral fish species. Proc Natl Acad Sci U S A. 2009;106: 1473–1478. doi:10.1073/pnas.0806804106

46. Milana V, Franchini P, Sola L, Angiulli E, Rossi AR. Genetic structure in lagoons: the effects of habitat discontinuity and low dispersal ability on populations of *Atherina boyeri*. Mar Biol. Springer-Verlag; 2012;159: 399–411. doi:10.1007/s00227-011-1817-1

47. Domingues VS, Bucciarelli G, Almada VC, Bernardi G. Historical colonization and demography of the Mediterranean damselfish, *Chromis chromis*. Mol Ecol. 2005;14: 4051–4063. doi:10.1111/j.1365-294X.2005.02723.x

48. Fruciano C, Hanel R, Debes P V., Tigano C, Ferrito V. Atlantic-Mediterranean and within-Mediterranean molecular variation in *Coris julis* (L. 1758) (Teleostei, Labridae). Mar Biol. Springer-Verlag; 2011;158: 1271–1286. doi:10.1007/s00227-011-1647-1

49. Francisco SM, Almada VC, Faria C, Velasco EM, Robalo JI. Phylogeographic pattern and glacial refugia of a rocky shore species with limited dispersal capability: the case of Montagu's blenny (*Coryphoblennius galerita*, Blenniidae). Mar Biol. Springer Berlin Heidelberg; 2014;161: 2509–2520. doi:10.1007/s00227-014-2523-6

50. Bahri-Sfar L, Lemaire C, Ben Hassine OK, Bonhomme F. Fragmentation of sea bass populations in the western and eastern Mediterranean as revealed by microsatellite polymorphism. Proc R Soc London Ser B-Biological Sci. 2000;267: 929–935.

51. Souche EL, Hellemans B, Babbucci M, Macaoidh E, Guinand B, Bargelloni L, et al. Range-wide population structure of European sea bass Dicentrarchus labrax. Biol J Linn Soc. 2015;116: 86–105. doi:10.1111/bij.12572

52. Kaoueche M, Bahri-Sfar L, Gonzalez-Wangueemert M, Perez-Ruzafa A, Ben Hassine OK. Allozyme and mtDNA variation of white seabream *Diplodus sargus* populations in a transition area between western and eastern Mediterranean basins (Siculo-Tunisian Strait). African J Mar Sci. Taylor & Francis Group; 2011;33: 79–90. doi:10.2989/1814232X.2011.572342

53. Magoulas A, Castilho R, Caetano S, Marcato S, Patarnello T. Mitochondrial DNA reveals a mosaic pattern of phylogeographical structure in Atlantic and Mediterranean populations of anchovy (*Engraulis encrasicolus*). Mol Phylogenet Evol. 2006;39: 734–746. doi:10.1016/j.ympev.2006.01.016

54. Schunter C, Carreras-Carbonell J, Planes S, Sala E, Ballesteros E, Zabala M, et

al. Genetic connectivity patterns in an endangered species: The dusky grouper (*Epinephelus marginatus*). J Exp Mar Bio Ecol. 2011;401: 126–133. doi:10.1016/j.jembe.2011.01.021

55. Milano I, Babbucci M, Cariani A, Atanassova M, Bekkevold D, Carvalho GR, et al. Outlier SNP markers reveal fine-scale genetic structuring across European hake populations (*Merluccius merluccius*). Mol Ecol. 2014;23: 118–135. doi:10.1111/mec.12568

56. Durand J, Blel H, Shen K, Koutrakis E, Guinand B. Population genetic structure of *Mugil cephalus* in the Mediterranean and Black Seas: a single mitochondrial clade and many nuclear barriers. Mar Ecol Prog Ser. 2013;474: 243–261. doi:10.3354/meps10080

57. Galarza JA, Turner GF, Macpherson E, Rico C. Patterns of genetic differentiation between two co-occurring demersal species: the red mullet (*Mullus barbatus*) and the striped red mullet (*Mullus surmuletus*). Can J Fish Aquat Sci. 2009;66: 1478–1490. doi:10.1139/f09-098

58. Mamuris Z, Apostolidis AP, Triantaphyllidis C. Genetic protein variation in red mullet (*Mullus barbatus*) and striped red mullet (*M. surmuletus*) populations from the Mediterranean Sea. Mar Biol. 1998;130: 353–360.

59. Maggio T, Lo Brutto S, Garoia F, Tinti F, Arculeo M. Microsatellite analysis of red mullet *Mullus barbatus* (Perciformes, Mullidae) reveals the isolation of the Adriatic Basin in the Mediterranean Sea. Ices J Mar Sci. 2009;66: 1883–1891. doi:10.1093/icesjms/fsp160

60. Félix-Hackradt FC, Hackradt CW, Pérez-Ruzafa Á, García-Charton JA. Discordant patterns of genetic connectivity between two sympatric species, *Mullus barbatus* (Linnaeus, 1758) and Mullus surmuletus (Linnaeus, 1758), in south-western Mediterranean Sea. Mar Environ Res. 2013;92: 23–34. doi:10.1016/j.marenvres.2013.08.008

61. Atarhouch T, Rami M, Naciri M, Dakkak A. Genetic population structure of sardine (*Sardina pilchardus*) off Morocco detected with intron polymorphism (EPIC-PCR). Mar Biol. 2007;150: 521–528. doi:10.1007/s00227-006-0371-8

62. Ramón MM, Castro JA. Genetic variation in natural stocks of *Sardina pilchardus* (sardines) from the western Mediterranean Sea. Heredity (Edinb). 1997;78: 520–528.

63. Ruggeri P, Splendiani A, Bonanomi S, Arneri E, Cingolani N, Santojanni A, et al. Searching for a stock structure in *Sardina pilchardus* from the Adriatic and Ionian seas using a microsatellite DNA-based approach. Sci Mar. 2013;77: 565–574. doi:10.3989/scimar.03843.26A

64. Boissin E, Micu D, Janczyszyn-Le Goff M, Neglia V, Bat L, Todorova V, et al. Contemporary genetic structure and postglacial demographic history of the black scorpionfish, *Scorpaena porcus*, in the Mediterranean and the Black Seas. Mol Ecol. 2016;33: 2195–2209. doi:10.1111/mec.13616

65. Kousteni V, Kasapidis P, Kotoulas G, Megalofonou P. Strong population genetic

structure and contrasting demographic histories for the small-spotted catshark (*Scyliorhinus canicula*) in the Mediterranean Sea. Heredity (Edinb). 2015;114: 333–43. doi:10.1038/hdy.2014.107

66. Šegvić-Bubić T, Marrone F, Grubišić L, Izquierdo-Gomez D, Katavić I, Arculeo M, et al. Two seas, two lineages: How genetic diversity is structured in Atlantic and Mediterranean greater amberjack *Seriola dumerili* Risso, 1810 (Perciformes, Carangidae). Fish Res. 2016;179: 271–279. doi:10.1016/j.fishres.2016.03.018

67. Schunter C, Carreras-Carbonell J, MacPherson E, Tintoré J, Vidal-Vijande E, Pascual A, et al. Matching genetics with oceanography: Directional gene flow in a Mediterranean fish species. Mol Ecol. 2011;20: 5167–5181. doi:10.1111/j.1365-294X.2011.05355.x

68. Guarniero I, Franzellitti S, Ungaro N, Tommasini S, Piccinetti C, Tinti F. Control region haplotype variation in the central Mediterranean common sole indicates geographical isolation and population structuring in Italian stocks. J Fish Biol. 2002;60: 1459–1474. doi:10.1006/jfbi.2002.2005

69. Kotoulas G, Bonhomme F, Borsa P. Genetic structure of the common sole *Solea vulgaris* at different geographic scales. Mar Biol. 1995;122: 361–375.

70. Kousteni V, Kasapidis P, Kotoulas G, Megalofonou P. Evidence of high genetic connectivity for the longnose spurdog *Squalus blainville* in the Mediterranean Sea. Mediterr Mar Sci. 2016;17: 371–383. doi:10.12681/mms.1222

71. Domingues VS, Alexandrou M, Almada VC, Robertson DR, Brito A, Santos RS, et al. Tropical fishes in a temperate sea: evolution of the wrasse *Thalassoma pavo* and the parrotfish *Sparisoma cretense* in the Mediterranean and the adjacent Macaronesian and Cape Verde Archipelagos. Mar Biol. 2008;154: 465–474. doi:10.1007/s00227-008-0941-z

72. Carreras-Carbonell J, Macpherson E, Pascual M. Population structure within and between subspecies of the Mediterranean triplefin fish *Tripterygion delaisi* revealed by highly polymorphic microsatellite loci. Mol Ecol. 2006;15: 3527–3539. doi:10.1111/j.1365-294X.2006.03003.x
