# Supplementary material for: Impact of life history traits on gene flow: A multispecies systematic review across oceanographic barriers in the Mediterranean Sea
Source: PLoS One. 2017 May 10;12(5):e0176419. doi: 10.1371/journal.pone.0176419 (PMC5425013; doi:10.1371/journal.pone.0176419)
Supplement: S2 Table — (PDF) [file pone.0176419.s003.pdf]

S2 Table. **Genetic and geographic distances between localities separated by fronts and no-fronts for the 176 datasets.** Species are classified by taxonomic group and the type of molecular marker used is specified. For each dataset we give the name of the localities and the genetic distance between them, as given in the References, for localities separated by a front and control localities without front between them. We have estimated approximate geographic distance between localities following the coastline with goggle earth.

				FRONT			NO-FRONT			
**Taxonomic group**	***Species***	**Marker**	**Front**	**Localities**	**Genetic distance**	**KM**	**Localities**	**Genetic distance**	**KM**	**References**
Angiosperm	*Cymodocea nodosa*	Microsatellites	GS	Ca-TC	0.229	183	TC-R	0.285	163	[1]
Angiosperm	*Cymodocea nodosa*	Microsatellites	AOF	R-U	0.142	253	TC-R	0.285	80	[1]
Angiosperm	*Cymodocea nodosa*	Microsatellites	IC	U-D	0.156	190	TC-R	0.285	80	[1]
Angiosperm	*Cymodocea nodosa*	Microsatellites	BF	F-Fa	0.174	259	TC-R	0.285	80	[1]
Angiosperm	*Cymodocea nodosa*	Microsatellites	ADR	CR-SM	0.423	1289	TC-R	0.285	80	[1]
Angiosperm	*Cymodocea nodosa*	Microsatellites	AEG	AN-CY	0.183	977	TC-R	0.285	80	[1]
Porifera	*Crambe crambe*	COI	IC	GAT-TOS	0.140	705	TOS-BNY	0.915	131	[2]
Porifera	*Crambe crambe*	COI	BF	BAL-TOS	0.060	313	TOS-BNY	0.915	131	[2]
Porifera	*Ircinia fasciculata*	Microsatellites	AOF	GRA-ALI	0.083	335	CAL-BLA	0.016	215	[3]
Porifera	*Ircinia fasciculata*	Microsatellites	IC	ALI-CAL	0.020	315	CAL-BLA	0.016	215	[3]
Porifera	*Ircinia fasciculata*	Microsatellites	BF	CAB-CAL	0.034	260	CAL-BLA	0.016	215	[3]

Group	*Species*	Marker	Front	Localities	Genetic	KM	Localities	Genetic	KM	References
Porifera	*Paraleucilla magna*	Microsatellites	GS	SGR-LHE	0.060	518	CGA-CPA	0.038	137	[4]
Porifera	*Paraleucilla magna*	Microsatellites	AOF	LHE-CGA	0.048	150	CGA-CPA	0.038	137	[4]
Porifera	*Paraleucilla magna*	Microsatellites	IC	BLN-CPA	0.057	536	CGA-CPA	0.038	137	[4]
Porifera	*Scopalina lophyropoda*	Microsatellites	IC	CG-MU	0.195	830	GA-MU	0.105	577	[5]
Porifera	*Spongia lamella*	Microsatellites	BF	Are-Cab	0.114	267	Are-Cr	0.047	130	[6]
Porifera	*Spongia officinalis*	Microsatellites	AEG	ECR-CYC	0.031	257	CYC-SPO	0.025	276	[7]
Cnidaria	*Astroides calycularis*	Microsatellites	AOF	GRA-PM	0.363	319	GRA-GAT	0.078	153	[8]
Cnidaria	*Aurelia aurita*	Allozymes	SC	BIZ-BB	0.025	577	SA-BB	0.003	217	[9]
Cnidaria	*Cladocora caespitosa*	Microsatellites	IC	IC-CP	0.028	273	LA-CI	0.027	110	[10]
Cnidaria	*Cladocora caespitosa*	Microsatellites	BF	IC-CG	0.051	267	LA-CI	0.027	110	[10]
Cnidaria	*Corallium rubrum*	Microsatellites	BF	FOR-XIC	0.072	237	FOR-LPP	0.034	83	[11]
Cnidaria	*Paramuricea clavata*	Microsatellites	AOF	TYL-CPS	0.115	501	MOU-ALT	0.124	473	[12]
Cnidaria	*Paramuricea clavata*	Microsatellites	IC	CPS-CLB	0.129	310	MOU-ALT	0.124	473	[12]
Mollusca	*Donax trunculus*	Microsatellites	GS	Don-Cala	0.018	250	Gan-SCRap	0.005	200	[13]
Mollusca	*Haliotis tuberculata*	Microsatellites	IC	CAR-BYS	0.031	624	AST-GAL	0.050	394	[14]
Mollusca	*Haliotis tuberculata*	Microsatellites	BF	BAL-BYS	0.037	273	AST-GAL	0.050	394	[14]

Group	*Species*	Marker	Front	Localities	Genetic	KM	Localities	Genetic	KM	References
Mollusca	*Mytilus galloprovincialis*	Microsatellites	GS	ATdo-ALma	0.021	294	ALma-ALsa	0.018	95	[15]
Mollusca	*Mytilus galloprovincialis*	Microsatellites	AOF	ALsa-MEse	0.027	190	ALma-ALsa	0.018	95	[15]
Mollusca	*Mytilus galloprovincialis*	Microsatellites	IC	MEse-MEcu	0.017	170	ALma-ALsa	0.018	95	[15]
Mollusca	*Octopus vulgaris*	COI	SC	GI-ZA	0.664	460	LI-ZA	0.743	230	[17]
Mollusca	*Octopus vulgaris*	COI	ADR	CRZ-PCS	0.005	500	PCS-STM	0.091	300	[16]
Mollusca	*Patella rustica*	Allozymes	GS	EST-CAS	0.050	439	PES-SAV	0.020	211	[18]
Mollusca	*Patella rustica*	COI	ADR	PES-CRT	0.350	736	PES-SAV	0.260	214	[18]
Mollusca	*Patella ulyssiponensis*	COI	GS	EST-CAD	0.375	230	CAD-CAS	0.161	230	[19]
Mollusca	*Patella ulyssiponensis*	COI	SC	CGA-PSE	-0.075	330	CAD-CAS	0.161	230	[19]
Mollusca	*Pinna nobilis*	COI	SC	N-M	-0.084	383	S-B	-0.087	161	[20]
Mollusca	*Pinna nobilis*	COI	ADR	VEN-PAC	0.400	1171	S-B	-0.087	161	[20]
Mollusca	*Pinna nobilis*	COI	AEG	XI-PAC	0.400	1173	S-B	-0.087	161	[20]
Mollusca	*Ruditapes decussatus*	COI	SC	MZJ-SFAX	0.238	350	SFAX-BIBEN	0.116	170	[21]
Mollusca	*Sepia officinalis*	Microsatellites	GS	Rmar-Faro	0.010	603	Lisb-Faro	0.000	327	[22]
Mollusca	*Sepia officinalis*	Microsatellites	AOF	Rmar-Alic	0.069	335	Lisb-Faro	0.000	327	[22]
Mollusca	*Sepia officinalis*	Microsatellites	IC	Alic-Vgel	0.009	490	Lisb-Faro	0.000	327	[22]

Group	*Species*	Marker	Front	Localities	Genetic	KM	Localities	Genetic	KM	References
Mollusca	*Sepia officinalis*	COI	BF	PAMA-VIGE	0.912	236	VIGE-SETE	0.095	392	[23]
Mollusca	*Sepia officinalis*	COI	SC	ALGI-GUHA	0.816	994	FARO-LISB	0.096	392	[23]
Mollusca	*Sepia officinalis*	COI	ADR	VENI-MALT	0.510	1419	FARO-LISB	0.096	392	[23]
Mollusca	*Sepia officinalis*	COI	AEG	AGNI-GUSI	0.474	753	FARO-LISB	0.096	392	[23]
Mollusca	*Solen marginatus*	Allozymes	SC	Biz-ElM	0.024	362	Biz-Rad	0.012	70	[24]
Crustacea	*Aristeomorpha foliacea*	ISSR	AEG	ION-AEG	0.033	650	PPA-ION	0.037	530	[25]
Crustacea	*Carcinus aestuarii*	Microsatellites	SC	CA-TA	0.028	1141	RA-FG	0.002	399	[26]
Crustacea	*Carcinus aestuarii*	Microsatellites	ADR	FG-TA	0.005	577	RA-FG	0.002	399	[26]
Crustacea	*Liocarcinus depurator*	COI	GS	CADIZ-MALAGA	0.061	218	VALENCIA-TARRAGONA	0.008	239	[27]
Crustacea	*Liocarcinus depurator*	COI	AOF	MALAGA-ALICANTE	0.015	499	VALENCIA-TARRAGONA	0.008	239	[27]
Crustacea	*Liocarcinus depurator*	COI	IC	ALICANTE-VALENCIA	0.124	191	VALENCIA-TARRAGONA	0.008	239	[27]
Crustacea	*Macropipus tuberculatus*	COI	GS	CADIZ-MALAGA	0.059	218	VALENCIA-TARRAGONA	0.015	239	[27]
Crustacea	*Macropipus tuberculatus*	COI	AOF	MALAGA-ALICANTE	0.035	499	VALENCIA-TARRAGONA	0.015	239	[27]
Crustacea	*Macropipus tuberculatus*	COI	IC	ALICANTE-VALENCIA	0.018	191	VALENCIA-TARRAGONA	0.015	239	[27]
Crustacea	*Munida intermedia*	COI	GS	CADIZ-MALAGA	0.044	218	VALENCIA-TARRAGONA	0.024	239	[27]
Crustacea	*Munida intermedia*	COI	AOF	MALAGA-ALICANTE	0.014	499	VALENCIA-TARRAGONA	0.024	239	[27]

Group	*Species*	Marker	Front	Localities	Genetic	KM	Localities	Genetic	KM	References
Crustacea	*Munida intermedia*	COI	IC	ALICANTE-VALENCIA	0.012	191	VALENCIA-TARRAGONA	0.024	239	[27]
Crustacea	*Pachygrapsus marmoratus*	COI	GS	MC-PS	0.003	705	VL-TA	0.080	211	[28]
Crustacea	*Pachygrapsus marmoratus*	COI	SC	BZ-SH	-0.036	250	VL-TA	0.080	211	[28]
Crustacea	*Pachygrapsus marmoratus*	COI	ADR	TG-PC	-0.102	200	VL-TA	0.080	211	[28]
Crustacea	*Pachygrapsus marmoratus*	COI	AEG	CH-OH	-0.030	590	VL-TA	0.080	211	[28]
Crustacea	*Pagurus alatus*	COI	GS	CADIZ-MALAGA	0.020	218	VALENCIA-TARRAGONA	0.000	239	[27]
Crustacea	*Pagurus alatus*	COI	AOF	MALAGA-ALICANTE	0.023	499	VALENCIA-TARRAGONA	0.000	239	[27]
Crustacea	*Pagurus alatus*	COI	IC	ALICANTE-VALENCIA	0.008	191	VALENCIA-TARRAGONA	0.000	239	[27]
Crustacea	*Pagurus excavatus*	COI	GS	CADIZ-MALAGA	0.030	218	VALENCIA-TARRAGONA	0.022	239	[27]
Crustacea	*Pagurus excavatus*	COI	AOF	MALAGA-ALICANTE	0.032	499	VALENCIA-TARRAGONA	0.022	239	[27]
Crustacea	*Pagurus excavatus*	COI	IC	ALICANTE-VALENCIA	0.022	191	VALENCIA-TARRAGONA	0.022	239	[27]
Crustacea	*Palaemon elegans*	16S-COI	GS	Gra-Cad	0.052	318	Gir-Ebro	0.021	252	[29]
Crustacea	*Palaemon elegans*	16S-COI	SC	Fus-Cal	-0.056	329	Gir-Ebro	0.021	252	[29]
Crustacea	*Palinurus elephas*	Microsatellites	BF	Columbretes-Mallorca	0.019	208	Mallorc-Menorca	0.001	223	[30]
Crustacea	*Parapenaeus longirostris*	COI	GS	CADIZ-MALAGA	0.023	218	VALENCIA-TARRAGONA	0.031	239	[27]
Crustacea	*Parapenaeus longirostris*	COI	AOF	MALAGA-ALICANTE	0.025	499	VALENCIA-TARRAGONA	0.031	239	[27]

Group	*Species*	Marker	Front	Localities	Genetic	KM	Localities	Genetic	KM	References
Crustacea	*Parapenaeus longirostris*	COI	IC	ALICANTE-VALENCIA	0.051	191	VALENCIA-TARRAGONA	0.031	239	[27]
Crustacea	*Parapenaeus longirostris*	D-Loop	SC	TYR1-SS1	0.011	352	SS1-SS2	0.018	311	[31]
Crustacea	*Parapenaeus longirostris*	D-Loop	ADR	AD1-SS2	-0.019	749	SS1-SS2	0.018	311	[31]
Crustacea	*Parapenaeus longirostris*	D-Loop	AEG	AEG2-SS2	0.252	1221	SS1-SS2	0.018	311	[31]
Crustacea	*Penaeus kerathurus*	COI	SC	MON-KAL	0.452	275	MON-GAB	-0.021	144	[32]
Crustacea	*Plesionika heterocarpus*	COI	GS	CADIZ-MALAGA	0.016	218	VALENCIA-TARRAGONA	0.015	239	[27]
Crustacea	*Plesionika heterocarpus*	COI	AOF	MALAGA-ALICANTE	0.036	499	VALENCIA-TARRAGONA	0.015	239	[27]
Crustacea	*Plesionika heterocarpus*	COI	IC	ALICANTE-VALENCIA	0.018	191	VALENCIA-TARRAGONA	0.015	239	[27]
Crustacea	*Stenosoma nadejda*	COI	GS	2 y 4	0.872	236	4 y 5	0.603	49	[33]
Crustacea	*Stenosoma nadejda*	COI	AOF	5 y 7	0.793	129	4 y 5	0.603	49	[33]
Echinodermata	*Arbacia lixula*	COI	AOF	HER-CAR	0.002	135	CAR-PAL	-0.014	150	[34]
Echinodermata	*Arbacia lixula*	COI	IC	BEN-CLM	0.015	171	CAR-PAL	-0.014	150	[34]
Echinodermata	*Arbacia lixula*	COI	BF	CLM-FOR	-0.026	142	CAR-PAL	-0.014	150	[34]
Echinodermata	*Astropecten aranciacus*	Microsatellites	GS	LAH-FAR	-0.001	464	GAE-MUR	-0.004	389	[35]
Echinodermata	*Echinaster sepisotus*	Microsatellites	IC	Pal-Bla	-0.031	508	Car-Pal	-0.025	157	[36]
Echinodermata	*Echinaster sepisotus*	Microsatellites	BF	Cab-Bla	0.030	262	Car-Pal	-0.025	157	[36]

Group	*Species*	Marker	Front	Localities	Genetic	KM	Localities	Genetic	KM	References
Echinodermata	*Echinaster sepisotus*	Microsatellites	SC	Tab-Mon	0.183	390	Car-Pal	-0.025	157	[36]
Echinodermata	*Echinaster sepisotus*	Microsatellites	ADR	Cro-Tao	0.115	874	Car-Pal	-0.025	157	[36]
Echinodermata	*Holothuria mammata*	COI	IC	GE-CP	-0.011	720	CP-ML	-0.010	415	[37]
Echinodermata	*Holothuria polii*	COI	IC	TH-GI	-0.020	510	MA-IS	-0.010	290	[39]
Echinodermata	*Holothuria polii*	COI	BF	GI-ML	0.004	255	MA-IS	-0.010	290	[39]
Echinodermata	*Holothuria polii*	Allozymes	SC	TAB-MON	0.050	390	MON-ZAR	0.049	280	[38]
Echinodermata	*Paracentrotus lividus*	COI	GS	CDr08-TFr08	-0.002	121	TS08-CL08	-0.002	200	[41]
Echinodermata	*Paracentrotus lividus*	COI	AOF	CBr08-TFr08	0.020	323	TS08-CL08	-0.002	200	[41]
Echinodermata	*Paracentrotus lividus*	COI	IC	CBr08-JVr08	0.028	355	TS08-CL08	-0.002	200	[41]
Echinodermata	*Paracentrotus lividus*	16S	BF	Cab-Tos	0.046	346	Col-Tos	0.003	281	[40]
Echinodermata	*Parastichopus regalis*	COI	AOF	AB-CG	0.047	205	CG-AL	-0.017	210	[42]
Echinodermata	*Parastichopus regalis*	COI	IC	AL-VAL	0.023	155	CG-AL	-0.017	210	[42]
Tunicata	*Pseudodistoma crucigaster*	COI	BF	Blanes-Cap Catalunya	0.929	191	Cadaqués-Blanes	0.416	61	[43]
Pisces	*Anguilla anguilla*	Microsatellites	GS	Oued Sebou-Moulonya	-0.001	544	Canet-Tour du Valat	0.001	184	[44]
Pisces	*Apogon imberbis*	Microsatellites	AOF	HE-CG	0.011	170	CG-MA	0.052	506	[45]
Pisces	*Apogon imberbis*	Microsatellites	IC	CG-BL	0.005	807	CG-MA	0.052	506	[45]

Group	*Species*	Marker	Front	Localities	Genetic	KM	Localities	Genetic	KM	References
Pisces	*Apogon imberbis*	Microsatellites	BF	MA-BL	0.060	294	CG-MA	0.052	506	[45]
Pisces	*Atherina boyeri*	Microsatellites	ADR	Ku-Bt	0.030	330	Ca-Ob	0.050	214	[46]
Pisces	*Chromis chromis*	D-Loop	SC	Pro/Nap-Goz	0.090	638	Orb-Nap	0.000	384	[47]
Pisces	*Chromis chromis*	D-Loop	AEG	Don-Goz	0.740	1122	Orb-Nap	0.000	384	[47]
Pisces	*Coris julis*	tRNA thr	BF	TM-ML	0.003	298	SP-CI	0.003	264	[48]
Pisces	*Coris julis*	tRNA thr	SC	NA-AU	0.012	799	SP-CI	0.003	264	[48]
Pisces	*Coris julis*	tRNA thr	ADR	LE-SP	0.011	524	SP-CI	0.003	264	[48]
Pisces	*Coris julis*	tRNA thr	AEG	GA-AU	0.029	925	SP-CI	0.003	264	[48]
Pisces	*Coryphoblennius galerita*	D-Loop	IC	CG-Ba	-0.026	795	Cad-Luz	0.169	291	[49]
Pisces	*Dicentrarchus labrax*	Microsatellites	GS	MKS1-MRBT	0.017	280	EGLV-FSET	0.017	680	[51]
Pisces	*Dicentrarchus labrax*	Microsatellites	AOF	MRBT-EMUR	0.056	590	EGLV-FSET	0.017	680	[51]
Pisces	*Dicentrarchus labrax*	Microsatellites	IC	EMUR-EGLV	0.017	320	EGLV-FSET	0.017	680	[51]
Pisces	*Dicentrarchus labrax*	Microsatellites	SC	TISK-TMAH	0.010	431	EGLV-FSET	-0.003	680	[50]
Pisces	*Dicentrarchus labrax*	Microsatellites	ADR	GMSL-ILDV	0.012	1305	EGLV-FSET	-0.003	680	[50]
Pisces	*Dicentrarchus labrax*	Microsatellites	AEG	YEGL-GTSK	0.050	1505	EGLV-FSET	-0.003	680	[50]
Pisces	*Diplodus sargus*	Allozymes	SC	BIZ-GA	0.026	460	MAH-GAB	0.035	170	[52]

Group	*Species*	Marker	Front	Localities	Genetic	KM	Localities	Genetic	KM	References
Pisces	*Diplodus vulgaris*	Microsatellites	AOF	HE-CG	0.060	170	CG-MA	0.001	506	[45]
Pisces	*Diplodus vulgaris*	Microsatellites	IC	CG-BL	0.008	807	CG-MA	0.001	506	[45]
Pisces	*Diplodus vulgaris*	Microsatellites	BF	MA-BL	0.004	294	CG-MA	0.001	506	[45]
Pisces	*Engraulis encrasicolus*	RFLPs	GS	Mal-Olh	0.017	401	Lio-Liv	0.005	736	[53]
Pisces	*Engraulis encrasicolus*	RFLPs	SC	Liv-Pat	0.007	1651	Lio-Liv	0.005	736	[53]
Pisces	*Engraulis encrasicolus*	RFLPs	ADR	Chi-Pat	0.032	1210	Lio-Liv	0.005	736	[53]
Pisces	*Engraulis encrasicolus*	RFLPs	AEG	Sar-Pat	0.004	495	Lio-Liv	0.005	736	[53]
Pisces	*Epinephelus marginatus*	Microsatellites	IC	CGA-BCN	0.002	641	BCN-MED	0.004	130	[54]
Pisces	*Epinephelus marginatus*	Microsatellites	BF	MALL-BCN	-0.003	208	BCN-MED	0.004	130	[54]
Pisces	*Epinephelus marginatus*	Microsatellites	SC	ALG-TUN	0.013	717	BCN-MED	0.004	130	[54]
Pisces	*Epinephelus marginatus*	Microsatellites	AEG	GRE-TUN	0.004	1237	BCN-MED	0.004	130	[54]
Pisces	*Merluccius merluccius*	SNPs	GS	NPT-MAL	0.006	230	NAD-SAD	-0.007	377	[55]
Pisces	*Merluccius merluccius*	SNPs	SC	STY-MAB	0.013	751	NAD-SAD	-0.007	377	[55]
Pisces	*Merluccius merluccius*	SNPs	ADR	SAD-NWJ	0.007	596	NAD-SAD	-0.007	377	[55]
Pisces	*Merluccius merluccius*	SNPs	AEG	AEG-CYP	0.001	1009	NAD-SAD	-0.007	377	[55]
Pisces	*Mugil cephalus*	Microsatellites	IC	WMA-WME	0.040	355	WME-WMT	0.003	494	[56]

Group	*Species*	Marker	Front	Localities	Genetic	KM	Localities	Genetic	KM	References
Pisces	*Mugil cephalus*	Microsatellites	SC	WMO-EMK	0.000	609	WME-WMT	0.003	494	[56]
Pisces	*Mugil cephalus*	Microsatellites	AEG	ASH-EMH	0.013	1322	WME-WMT	0.003	494	[56]
Pisces	*Mullus barbatus*	Microsatellites	AOF	FU-GA	-0.001	260	FU-AL	0.002	230	[60]
Pisces	*Mullus barbatus*	Microsatellites	IC	CG-BL	0.022	862	BL-IT	0.019	729	[57]
Pisces	*Mullus barbatus*	Microsatellites	SC	TIR5-SS3	0.008	320	IO1-SS3	0.006	100	[59]
Pisces	*Mullus barbatus*	Allozymes	AEG	4 y 5	0.001	1235	4 y 3	0.002	132	[58]
Pisces	*Mullus surmuletus*	Microsatellites	AOF	HE-CG	0.021	170	CG-MA	0.127	506	[45]
Pisces	*Mullus surmuletus*	Microsatellites	IC	CG-BL	0.001	862	CG-MA	0.127	506	[45]
Pisces	*Mullus surmuletus*	Microsatellites	BF	MA-BL	0.134	294	CG-MA	0.127	506	[45]
Pisces	*Oblada melanura*	Microsatellites	AOF	HE-CG	0.001	170	CG-MA	0.155	506	[45]
Pisces	*Oblada melanura*	Microsatellites	IC	CG-BL	0.052	807	CG-MA	0.155	506	[45]
Pisces	*Oblada melanura*	Microsatellites	BF	MA-BL	0.170	294	CG-MA	0.155	506	[45]
Pisces	*Sardina pilchardus*	EPIC	GS	Larache-Nador	0.034	416	Essaouira-Larache	0.002	319	[61]
Pisces	*Sardina pilchardus*	Allozymes	AOF	1 y 2	0.051	217	6 y 8	0.048	353	[62]
Pisces	*Sardina pilchardus*	Allozymes	IC	2 y 5	0.060	305	6 y 8	0.048	353	[62]
Pisces	*Sardina pilchardus*	Microsatellites	ADR	TR-IO	0.002	805	CH-SB	-0.001	346	[63]

Group	*Species*	Marker	Front	Localities	Genetic	KM	Localities	Genetic	KM	References
Pisces	*Scorpaena porcus*	Microsatellites	ADR	PC-TG	0.007	200	TG-TRE	0.008	272	[64]
Pisces	*Scyliorhinus canicula*	Microsatellites	AEG	ION-CRE	0.025	585	CRE-MYR	0.004	254	[65]
Pisces	*Seriola dumerilli*	D-loop	SC	TU-SR	0.031	530	TU-SA	-0.041	400	[66]
Pisces	*Serranus cabrilla*	Microsatellites	AOF	HE-CG	0.013	174	BL-CC	-0.002	120	[67]
Pisces	*Serranus cabrilla*	Microsatellites	IC	CP-C	0.044	386	BL-CC	-0.002	120	[67]
Pisces	*Serranus cabrilla*	Microsatellites	BF	MA-BL	0.008	221	BL-CC	-0.002	120	[67]
Pisces	*Serranus cabrilla*	Microsatellites	SC	CER-OT	0.000	1224	BL-CC	-0.002	120	[67]
Pisces	*Serranus cabrilla*	Microsatellites	AEG	GR-OT	0.002	888	BL-CC	-0.002	120	[67]
Pisces	*Solea vulgaris*	D-Loop	SC	SAL-SIC	0.043	789	LIV-SAL	-0.007	624	[68]
Pisces	*Solea vulgaris*	D-Loop	ADR	NER-GAL	0.024	437	LIV-SAL	-0.007	624	[68]
Pisces	*Solea vulgaris*	Allozymes	AEG	GR-EG	0.013	1308	E1-M1	0.007	458	[69]
Pisces	*Squalus blainville*	COI	SC	SIC-MAL	0.029	300	MAL-ION	-0.065	590	[70]
Pisces	*Squalus blainville*	COI	AEG	CRE-CYC	-0.278	292	MAL-ION	-0.065	590	[70]
Pisces	*Symphodus tinca*	Microsatellites	AOF	HE-CG	0.033	170	CG-MA	0.001	506	[45]
Pisces	*Symphodus tinca*	Microsatellites	IC	CG-BL	0.001	807	CG-MA	0.001	506	[45]
Pisces	*Symphodus tinca*	Microsatellites	BF	MA-BL	0.002	294	CG-MA	0.001	506	[45]

**Group**	***Species***	**Marker**	**Front**	**Localities**	**Genetic**	**KM**	**Localities**	**Genetic**	**KM**	**References**
Pisces	*Thalassoma pavo*	D-Loop	SC	Ust-Lin	0.000	416	Gen-Poz	0.000	736	[71]
Pisces	*Thalassoma pavo*	D-Loop	AEG	Kea-lin	0.571	1168	Gen-Poz	0.000	736	[71]
Pisces	*Tripterigion delaisi*	Microsatellites	AOF	TA-GA	0.046	293	CO-BL	0.029	263	[72]
Pisces	*Tripterigion delaisi*	Microsatellites	IC	PA-CO	0.036	386	CO-BL	0.029	263	[72]
Pisces	*Tripterigion delaisi*	Microsatellites	BF	CO-FO	0.010	167	CO-BL	0.029	263	[72]

References

1. Alberto F, Massa S, Manent P, Diaz-Almela E, Arnaud-Haond S, Duarte CM, et al. Genetic differentiation and secondary contact zone in the seagrass *Cymodocea nodosa* across the Mediterranean-Atlantic transition region. J Biogeogr. 2008;35: 1279–1294. doi:10.1111/j.1365-2699.2007.01876.x

2. Duran S, Pascual M, Turon X. Low levels of genetic variation in mtDNA sequences over the western Mediterranean and Atlantic range of the sponge *Crambe crambe* (Poecilosclerida). Mar Biol. 2004;144: 31–35. doi:10.1007/s00227-003-1178-5

3. Riesgo A, Pérez-Portela R, Pita L, Blasco G, Erwin PM, López-Legentil S. Population structure and connectivity in the Mediterranean sponge Ircinia fasciculata are affected by mass mortalities and hybridization. Heredity (Edinb). Nature Publishing Group; 2016;117: 1–13. doi:10.1038/hdy.2016.41

4. Guardiola M, Frotscher J, Uriz MJ. High genetic diversity, phenotypic plasticity, and invasive potential of a recently introduced calcareous sponge, fast spreading across the Atlanto-Mediterranean basin. Mar Biol. Springer Berlin Heidelberg; 2016;163: 123. doi:10.1007/s00227-016-2862-6

5. Blanquer A, Uriz MJ. Population genetics at three spatial scales of a rare sponge living in fragmented habitats. Bmc Evol Biol. 2010;10. doi:1310.1186/1471-2148-10-13

6. Pérez-Portela R, Noyer C, Becerro MA. Genetic structure and diversity of the endangered bath sponge *Spongia lamella*. Aquat Conserv Mar Freshw Ecosyst. 2015;25: 365–379. doi:10.1002/aqc.2423

7. Dailianis T, Tsigenopoulos CS, Dounas C, Voultsiadou E. Genetic diversity of the imperilled bath sponge *Spongia officinalis* Linnaeus, 1759 across the Mediterranean Sea: Patterns of population differentiation and implications for taxonomy and conservation. Mol Ecol. Blackwell Publishing Ltd; 2011;20: 3757–3772. doi:10.1111/j.1365-294X.2011.05222.x

8. Casado-Amezúa P, Goffredo S, Templado J, Machordom A. Genetic assessment of population structure and connectivity in the threatened mediterranean coral *Astroides calycularis* (Scleractinia, Dendrophylliidae) at different spatial scales. Mol Ecol. 2012;21: 3671–3685. doi:10.1111/j.1365-294X.2012.05655.x

9. Ben Faleh A, Ben Othmen A, Deli T, Annabi A, Said K. High genetic homogeneity of the moon jelly *Aurelia aurita* (Scyphozoa, Semaeostomeae) along the Mediterranean coast of Tunisia. African J Mar Sci. Taylor & Francis Group; 2009;31: 73–80. doi:10.2989/AJMS.2009.31.1.6.777

10. Casado-Amezúa P, Kersting DK, Templado J, Machordom A. Regional genetic differentiation among populations of *Cladocora caespitosa* in the Western Mediterranean. Coral Reefs. Springer Berlin Heidelberg; 2014;33: 1031–1040. doi:10.1007/s00338-014-1195-5

11. Ledoux JB, Mokhtar-Jamai K, Roby C, Feral JP, Garrabou J, Aurelle D. Genetic survey of shallow populations of the Mediterranean red coral *Corallium rubrum*

(Linnaeus, 1758) : new insights into evolutionary processes shaping nuclear diversity and implications for conservation. Mol Ecol. 2010;19: 675–690. doi:10.1111/j.1365-294X.2009.04516.x

12. Mokhtar-Jamaï K, Pascual M, Ledoux JB, Coma R, Féral JP, Garrabou J, et al. From global to local genetic structuring in the red gorgonian *Paramuricea clavata*: The interplay between oceanographic conditions and limited larval dispersal. Mol Ecol. 2011;20: 3291–3305. doi:10.1111/j.1365-294X.2011.05176.x

13. Marie AD, Lejeusne C, Karapatsiou E, Cuesta JA, Drake P, Macpherson E, et al. Implications for management and conservation of the population genetic structure of the wedge clam Donax trunculus across two biogeographic boundaries. Sci Rep. 2016;6. doi:10.1038/srep39152

14. Roussel V, van Wormhoudt A. The Effect of Pleistocene Climate Fluctuations on Distribution of European Abalone (*Haliotis tuberculata*), Revealed by Combined Mitochondrial and Nuclear Marker Analyses. Biochemical Genetics. Springer US; 20 Oct 2016: 1–31. doi:10.1007/s10528-016-9778-1

15. Diz AP, Presa P. Regional patterns of microsatellite variation in Mytilus galloprovincialis from the Iberian Peninsula. Mar Biol. 2008;154: 277–286. doi:10.1007/s00227-008-0921-3

16. De Luca D, Catanese G, Procaccini G, Fiorito G. *Octopus vulgaris* (Cuvier, 1797) in the Mediterranean Sea: Genetic diversity and population structure. Zane L, editor. PLoS One. Public Library of Science; 2016;11: e0149496. doi:10.1371/journal.pone.0149496

17. Fadhlaoui-Zid K, Knittweis L, Aurelle D, Nafkha C, Ezzeddine S, Fiorentino F, et al. Genetic structure of *Octopus vulgaris* (Cephalopoda, Octopodidae) in the central Mediterranean Sea inferred from the mitochondrial COIII gene. Comptes Rendus - Biol. 2012;335: 625–636. doi:10.1016/j.crvi.2012.10.004

18. Sá-Pinto A, Branco MS, Alexandrino PB, Fontaine MC, Baird SJE. Barriers to Gene Flow in the Marine Environment: Insights from Two Common Intertidal Limpet Species of the Atlantic and Mediterranean. Steinke D, editor. PLoS One. Public Library of Science; 2012;7: e50330. doi:10.1371/journal.pone.0050330

19. Cossu P, Dedola GL, Scarpa F, Sanna D, Lai T, Maltagliati F, et al. Patterns of spatial genetic variation in *Patella ulyssiponensis*: insights from the western Mediterranean marine ecoregion. Hydrobiologia. 2015;755: 39–55. doi:10.1007/s10750-015-2216-2

20. Sanna D, Cossu P, Dedola GL, Scarpa F, Maltagliati F, Castelli A, et al. Mitochondrial DNA Reveals Genetic Structuring of *Pinna nobilis* across the Mediterranean Sea. Colgan DJ, editor. PLoS One. Public Library of Science; 2013;8: e67372. doi:10.1371/journal.pone.0067372

21. Gharbi A, Chatti N, Said K, Wormhoudt A. Genetic variation and population structure of the carpet shell clam *Ruditapes decussatus* along the Tunisian coast inferred from mtDNA and ITS1 sequence analysis. Biologia (Bratisl). SP Versita; 2010;65: 688–696. doi:10.2478/s11756-010-0069-8

22. Pérez-Losada M, Guerra A, Carvalho GR, Sanjuan A, Shaw PW. Extensive population subdivision of the cuttlefish *Sepia officinalis* (Mollusca : Cephalopoda) around the Iberian Peninsula indicated by microsatellite DNA variation. Heredity (Edinb). 2002;89: 417–424. doi:10.1038/sj.hdy.6800160

23. Pérez-Losada M, Nolte MJ, Crandall KA, Shaw PW. Testing hypotheses of population structuring in the Northeast Atlantic Ocean and Mediterranean Sea using the common cuttlefish *Sepia officinalis*. Mol Ecol. 2007;16: 2667–2679. doi:10.1111/j.1365-294X.2007.03333.x

24. Hmida L, Fassatoui C, Ayed D, Ayache N, Romdhane MS. Genetic characterization of the razor clam *Solen marginatus* (Mollusca: Bivalvia: Solenidae) in Tunisian coasts based on isozyme markers. Biochem Syst Ecol. 2012;40: 146–155. doi:10.1016/j.bse.2011.10.016

25. Fernández MV, Maltagliati F, Pannacciulli FG, Roldán MI. Analysis of genetic variability in *Aristaeomorpha foliacea* (Crustacea, Aristeidae) using DNA-ISSR (Inter Simple Sequence Repeats) markers. Comptes Rendus - Biol. 2011;334: 705–712. doi:10.1016/j.crvi.2011.07.005

26. Schiavina M, Marino IAM, Zane L, Melià P. Matching oceanography and genetics at the basin scale. Seascape connectivity of the Mediterranean shore crab in the Adriatic Sea. Mol Ecol. 2014;23: 5496–5507. doi:10.1111/mec.12956

27. García-Merchán VH, Robainas-Barcia A, Abelló P, Macpherson E, Palero F, García-Rodríguez M, et al. Phylogeographic patterns of decapod crustaceans at the Atlantic–Mediterranean transition. Mol Phylogenet Evol. 2012;62: 664–672. doi:10.1016/j.ympev.2011.11.009

28. Fratini S, Ragionieri L, Deli T, Harrer A, Marino IAM, Cannicci S, et al. Unravelling population genetic structure with mitochondrial DNA in a notional panmictic coastal crab species: sample size makes the difference. BMC Evol Biol. 2016;16: 150. doi:10.1186/s12862-016-0720-2

29. Reuschel S, Cuesta JA, Schubart CD. Marine biogeographic boundaries and human introduction along the European coast revealed by phylogeography of the prawn *Palaemon elegans*. Mol Phylogenet Evol. 2010;55: 765–775. doi:10.1016/j.ympev.2010.03.021

30. Babbucci M, Buccoli S, Cau A, Cannas R, Goñi R, Díaz D, et al. Population structure, demographic history, and selective processes: Contrasting evidences from mitochondrial and nuclear markers in the European spiny lobster *Palinurus elephas* (Fabricius, 1787). Mol Phylogenet Evol. 2010;56: 1040–1050. doi:10.1016/j.ympev.2010.05.014

31. Lo Brutto S, Maggio T, Arculeo M. Isolation By Distance (IBD) signals in the deep-water rose shrimp *Parapenaeus longirostris* (Lucas, 1846) (Decapoda, Panaeidae) in the Mediterranean Sea. Mar Environ Res. 2013;90: 1–8. doi:10.1016/j.marenvres.2013.05.006

32. Zitari-Chatti R, Chatti N, Fulgione D, Caiazza I, Aprea G, Elouaer A, et al. Mitochondrial DNA variation in the caramote prawn *Penaeus (Melicertus) kerathurus* across a transition zone in the Mediterranean Sea. Genetica.

2009;136: 439–447. doi:10.1007/s10709-008-9344-9

33. Xavier R, Zenboudji S, Lima FP, Harris DJ, Santos AM, Branco M. Phylogeography of the marine isopod *Stenosoma nadejda* (Rezig, 1989) in North African Atlantic and western Mediterranean coasts reveals complex differentiation patterns and a new species. Biol J Linn Soc. Blackwell Publishing Ltd; 2011;104: 419–431. doi:10.1111/j.1095-8312.2011.01718.x

34. Wangensteen OS, Turon X, Pérez-Portela R, Palacín C. Natural or Naturalized? Phylogeography Suggests That the Abundant Sea Urchin *Arbacia lixula* Is a Recent Colonizer of the Mediterranean. Dupont S, editor. PLoS One. Public Library of Science; 2012;7: e45067. doi:10.1371/journal.pone.0045067

35. Zulliger DE, Tanner S, Ruch M, Ribi G. Genetic structure of the high dispersal Atlanto-Mediterreanean sea star *Astropecten aranciacus* revealed by mitochondrial DNA sequences and microsatellite loci. Mar Biol. 2009;156: 597–610. doi:10.1007/s00227-008-1111-z

36. Garcia-Cisneros A, Palacín C, Ben Khadra Y, Pérez-Portela R. Low genetic diversity and recent demographic expansion in the red starfish *Echinaster sepositus* (Retzius 1816). Sci Rep. Nature Publishing Group; 2016;6: 33269. doi:10.1038/srep33269

37. Borrero-Pérez GH, González-Wangüemert M, Marcos C, Pérez-Ruzafa A. Phylogeography of the Atlanto-Mediterranean sea cucumber *Holothuria (Holothuria) mammata*: The combined effects of historical processes and current oceanographical pattern. Mol Ecol. 2011;20: 1964–1975. doi:10.1111/j.1365-294X.2011.05068.x

38. Gharbi A, Said K. Genetic variation and population structure of *Holothuria polii* from the eastern and western Mediterranean coasts in Tunisia. J Mar Biol Assoc United Kingdom. Cambridge University Press; 2011;91: 1599–1606. doi:10.1017/S0025315411000245

39. Valente S, Serrão EA, González-Wangüemert M. West versus East Mediterranean Sea: origin and genetic differentiation of the sea cucumber *Holothuria polii*. Mar Ecol. 2015;36: 485–495. doi:10.1111/maec.12156

40. Calderón I, Giribet G, Turon X. Two markers and one history: phylogeography of the edible common sea urchin *Paracentrotus lividus* in the Lusitanian region. Mar Biol. 2008;154: 137–151. doi:10.1007/s00227-008-0908-0

41. Calderón I, Pita L, Brusciotti S, Palacín C, Turon X. Time and space: genetic structure of the cohorts of the common sea urchin *Paracentrotus lividus* in Western Mediterranean. Mar Biol. 2012;159: 187–197. doi:10.1007/s00227-011-1799-z

42. Maggi C, González-Wangüemert M. Genetic differentiation among *Parastichopus regalis* populations in the Western Mediterranean Sea: Potential effects from its fishery and current connectivity. Mediterr Mar Sci. 2015;16: 489–501. doi:10.12681/mms.1020

43. Tarjuelo I, Posada D, Crandall KA, Pascual M, Turon X. Phylogeography and

speciation of colour morphs in the colonial ascidian *Pseudodistoma crucigaster*. Mol Ecol. 2004;13: 3125–3136. doi:10.1111/j.1365-294X.2004.02306.x

44. Als TD, Hansen MM, Maes GE, Castonguay M, Riemann L, Aarestrup K, et al. All roads lead to home: Panmixia of European eel in the Sargasso Sea. Mol Ecol. 2011;20: 1333–1346. doi:10.1111/j.1365-294X.2011.05011.x

45. Galarza JA, Carreras-Carbonell J, Macpherson E, Pascual M, Roques S, Turner GF, et al. The influence of oceanographic fronts and early-life-history traits on connectivity among littoral fish species. Proc Natl Acad Sci U S A. 2009;106: 1473–1478. doi:10.1073/pnas.0806804106

46. Milana V, Franchini P, Sola L, Angiulli E, Rossi AR. Genetic structure in lagoons: the effects of habitat discontinuity and low dispersal ability on populations of *Atherina boyeri*. Mar Biol. Springer-Verlag; 2012;159: 399–411. doi:10.1007/s00227-011-1817-1

47. Domingues VS, Bucciarelli G, Almada VC, Bernardi G. Historical colonization and demography of the Mediterranean damselfish, *Chromis chromis*. Mol Ecol. 2005;14: 4051–4063. doi:10.1111/j.1365-294X.2005.02723.x

48. Fruciano C, Hanel R, Debes P V., Tigano C, Ferrito V. Atlantic-Mediterranean and within-Mediterranean molecular variation in *Coris julis* (L. 1758) (Teleostei, Labridae). Mar Biol. Springer-Verlag; 2011;158: 1271–1286. doi:10.1007/s00227-011-1647-1

49. Francisco SM, Almada VC, Faria C, Velasco EM, Robalo JI. Phylogeographic pattern and glacial refugia of a rocky shore species with limited dispersal capability: the case of Montagu's blenny (*Coryphoblennius galerita*, Blenniidae). Mar Biol. Springer Berlin Heidelberg; 2014;161: 2509–2520. doi:10.1007/s00227-014-2523-6

50. Bahri-Sfar L, Lemaire C, Ben Hassine OK, Bonhomme F. Fragmentation of sea bass populations in the western and eastern Mediterranean as revealed by microsatellite polymorphism. Proc R Soc London Ser B-Biological Sci. 2000;267: 929–935.

51. Souche EL, Hellemans B, Babbucci M, Macaoidh E, Guinand B, Bargelloni L, et al. Range-wide population structure of European sea bass Dicentrarchus labrax. Biol J Linn Soc. 2015;116: 86–105. doi:10.1111/bij.12572

52. Kaoueche M, Bahri-Sfar L, Gonzalez-Wangueemert M, Perez-Ruzafa A, Ben Hassine OK. Allozyme and mtDNA variation of white seabream *Diplodus sargus* populations in a transition area between western and eastern Mediterranean basins (Siculo-Tunisian Strait). African J Mar Sci. Taylor & Francis Group; 2011;33: 79–90. doi:10.2989/1814232X.2011.572342

53. Magoulas A, Castilho R, Caetano S, Marcato S, Patarnello T. Mitochondrial DNA reveals a mosaic pattern of phylogeographical structure in Atlantic and Mediterranean populations of anchovy (*Engraulis encrasicolus*). Mol Phylogenet Evol. 2006;39: 734–746. doi:10.1016/j.ympev.2006.01.016

54. Schunter C, Carreras-Carbonell J, Planes S, Sala E, Ballesteros E, Zabala M, et

al. Genetic connectivity patterns in an endangered species: The dusky grouper (*Epinephelus marginatus*). J Exp Mar Bio Ecol. 2011;401: 126–133. doi:10.1016/j.jembe.2011.01.021

55. Milano I, Babbucci M, Cariani A, Atanassova M, Bekkevold D, Carvalho GR, et al. Outlier SNP markers reveal fine-scale genetic structuring across European hake populations (*Merluccius merluccius*). Mol Ecol. 2014;23: 118–135. doi:10.1111/mec.12568

56. Durand J, Blel H, Shen K, Koutrakis E, Guinand B. Population genetic structure of *Mugil cephalus* in the Mediterranean and Black Seas: a single mitochondrial clade and many nuclear barriers. Mar Ecol Prog Ser. 2013;474: 243–261. doi:10.3354/meps10080

57. Galarza JA, Turner GF, Macpherson E, Rico C. Patterns of genetic differentiation between two co-occurring demersal species: the red mullet (*Mullus barbatus*) and the striped red mullet (*Mullus surmuletus*). Can J Fish Aquat Sci. 2009;66: 1478–1490. doi:10.1139/f09-098

58. Mamuris Z, Apostolidis AP, Triantaphyllidis C. Genetic protein variation in red mullet (*Mullus barbatus*) and striped red mullet (*M. surmuletus*) populations from the Mediterranean Sea. Mar Biol. 1998;130: 353–360.

59. Maggio T, Lo Brutto S, Garoia F, Tinti F, Arculeo M. Microsatellite analysis of red mullet *Mullus barbatus* (Perciformes, Mullidae) reveals the isolation of the Adriatic Basin in the Mediterranean Sea. Ices J Mar Sci. 2009;66: 1883–1891. doi:10.1093/icesjms/fsp160

60. Félix-Hackradt FC, Hackradt CW, Pérez-Ruzafa Á, García-Charton JA. Discordant patterns of genetic connectivity between two sympatric species, *Mullus barbatus* (Linnaeus, 1758) and Mullus surmuletus (Linnaeus, 1758), in south-western Mediterranean Sea. Mar Environ Res. 2013;92: 23–34. doi:10.1016/j.marenvres.2013.08.008

61. Atarhouch T, Rami M, Naciri M, Dakkak A. Genetic population structure of sardine (*Sardina pilchardus*) off Morocco detected with intron polymorphism (EPIC-PCR). Mar Biol. 2007;150: 521–528. doi:10.1007/s00227-006-0371-8

62. Ramón MM, Castro JA. Genetic variation in natural stocks of *Sardina pilchardus* (sardines) from the western Mediterranean Sea. Heredity (Edinb). 1997;78: 520–528.

63. Ruggeri P, Splendiani A, Bonanomi S, Arneri E, Cingolani N, Santojanni A, et al. Searching for a stock structure in *Sardina pilchardus* from the Adriatic and Ionian seas using a microsatellite DNA-based approach. Sci Mar. 2013;77: 565–574. doi:10.3989/scimar.03843.26A

64. Boissin E, Micu D, Janczyszyn-Le Goff M, Neglia V, Bat L, Todorova V, et al. Contemporary genetic structure and postglacial demographic history of the black scorpionfish, *Scorpaena porcus*, in the Mediterranean and the Black Seas. Mol Ecol. 2016;33: 2195–2209. doi:10.1111/mec.13616

65. Kousteni V, Kasapidis P, Kotoulas G, Megalofonou P. Strong population genetic

structure and contrasting demographic histories for the small-spotted catshark (*Scyliorhinus canicula*) in the Mediterranean Sea. Heredity (Edinb). 2015;114: 333–43. doi:10.1038/hdy.2014.107

66. Šegvić-Bubić T, Marrone F, Grubišić L, Izquierdo-Gomez D, Katavić I, Arculeo M, et al. Two seas, two lineages: How genetic diversity is structured in Atlantic and Mediterranean greater amberjack *Seriola dumerili* Risso, 1810 (Perciformes, Carangidae). Fish Res. 2016;179: 271–279. doi:10.1016/j.fishres.2016.03.018

67. Schunter C, Carreras-Carbonell J, MacPherson E, Tintoré J, Vidal-Vijande E, Pascual A, et al. Matching genetics with oceanography: Directional gene flow in a Mediterranean fish species. Mol Ecol. 2011;20: 5167–5181. doi:10.1111/j.1365-294X.2011.05355.x

68. Guarniero I, Franzellitti S, Ungaro N, Tommasini S, Piccinetti C, Tinti F. Control region haplotype variation in the central Mediterranean common sole indicates geographical isolation and population structuring in Italian stocks. J Fish Biol. 2002;60: 1459–1474. doi:10.1006/jfbi.2002.2005

69. Kotoulas G, Bonhomme F, Borsa P. Genetic structure of the common sole *Solea vulgaris* at different geographic scales. Mar Biol. 1995;122: 361–375.

70. Kousteni V, Kasapidis P, Kotoulas G, Megalofonou P. Evidence of high genetic connectivity for the longnose spurdog *Squalus blainville* in the Mediterranean Sea. Mediterr Mar Sci. 2016;17: 371–383. doi:10.12681/mms.1222

71. Domingues VS, Alexandrou M, Almada VC, Robertson DR, Brito A, Santos RS, et al. Tropical fishes in a temperate sea: evolution of the wrasse *Thalassoma pavo* and the parrotfish *Sparisoma cretense* in the Mediterranean and the adjacent Macaronesian and Cape Verde Archipelagos. Mar Biol. 2008;154: 465–474. doi:10.1007/s00227-008-0941-z

72. Carreras-Carbonell J, Macpherson E, Pascual M. Population structure within and between subspecies of the Mediterranean triplefin fish *Tripterygion delaisi* revealed by highly polymorphic microsatellite loci. Mol Ecol. 2006;15: 3527–3539. doi:10.1111/j.1365-294X.2006.03003.x
