# Supplementary material for: Impact of life history traits on gene flow: A multispecies systematic review across oceanographic barriers in the Mediterranean Sea
Source: PLoS One. 2017 May 10;12(5):e0176419. doi: 10.1371/journal.pone.0176419 (PMC5425013; doi:10.1371/journal.pone.0176419)
Supplement: S3 Table — (PDF) [file pone.0176419.s004.pdf]

Table S3. **Number of species, for each PLD category and LIFE strategy, showing Non significant/Significant genetic distances and No reduction/ Reduction gene flow between localities separated by each oceanographic front.** PLD categories: S=1-15 days, M=16-30 days, L≥31 days. LIFE strategies: BS=benthic sessile or limited motility, BM=benthic vagile, PEL=pelagic. The fronts analysed are: Gibraltar Strait (GS), Almeria-Oran Front (AOF), Ibiza Channel (IC), Balearic Front (BF), Sicily Channel (SC), Otranto Channel (ADR), and the southern margin of the Aegean Sea (AEG).

			**GS**	**AOF**	**IC**	**BF**	**SC**	**ADR**	**AEG**
PLD	L	Non significant	7	3	8	4	11	8	8
		Significant	4	5	4	2	3	0	2
		No reduction	3	3	3	1	8	2	3
		Reduction	8	5	9	5	6	6	7
	M	Non significant	5	8	10	4	4	2	3
		Significant	5	4	4	3	6	2	1
		No reduction	2	6	7	3	5	1	2
		Reduction	8	6	7	4	5	3	2
	S	Non significant	0	1	4	4	3	3	1
		Significant	4	7	6	6	1	2	5
		No reduction	3	3	7	5	1	3	2
		Reduction	1	5	3	5	3	2	4
LIFE	PEL	Non significant	1	1	1	0	4	3	2
		Significant	3	2	3	1	1	0	1
		No reduction	0	1	1	0	2	0	0
		Reduction	4	2	3	1	3	3	3
	BM	Non significant	9	8	12	6	11	9	10
		Significant	4	8	6	5	7	2	4
		No reduction	4	8	8	3	8	4	6
		Reduction	9	8	10	8	10	7	8
	BS	Non significant	2	3	9	6	4	1	0
		Significant	6	6	5	5	1	2	3
		No reduction	4	3	8	6	4	2	1
		Reduction	4	6	6	5	1	1	2
